# Psychometric properties and contextual appropriateness of the German version of the Early Development Instrument

**DOI:** 10.1186/s12887-020-02191-w

**Published:** 2020-07-09

**Authors:** Sabine Georg, Catherin Bosle, Joachim E. Fischer, Freia De Bock

**Affiliations:** 1 Mannheim Institute of Public Health, Social and Preventive Medicin, Medical Faculty Mannheim of the Ruprecht-Karls-University Heidelberg, Ludolf-Krehl-Straße 7-11, 68167 Mannheim, Germany; 2 Federal Centre for Health Education, Maarweg 149-161, 50625 Cologne, Germany

**Keywords:** Child development, Early Development Instrument, Germany, Validity, Public health planning tool

## Abstract

**Background:**

Assessing the early development of children at a population level in educational settings, may be useful for public health and policy decision making. In this study, we evaluated the psychometric properties and the contextual appropriateness of a German language version of the Early Development Instrument (EDI), a survey-based instrument originally developed in Canada, which assesses developmental vulnerability for children in preschool settings.

**Methods:**

Sixty preschool teachers from six preschool organizations (22% of organizations contacted) in three cities in southwest Germany participated. They administered a German version of the EDI (GEDI) to 225 children (51% of eligible children). We assessed internal consistency, test-retest and interrater reliability. Preschool teachers assisted in determining face-validity by reviewing item coverage and comprehensibility. Exploratory factor analysis (EFA) was used to evaluate convergent validity. Concurrent validity was measured using correlations and agreements (Bland-Altman plots) between GEDI and other validated instrument scores. Additionally, we compared associations between GEDI domain scores and sociodemographic characteristics with similar associations in EDI studies worldwide.

**Results:**

GEDI domains showed good to excellent internal consistency (0.73 < α > 0.99) and moderate to good test-retest and interrater reliability (0.50 to 0.81 and 0.48 to 0.71, respectively [*p*-value < 0.05]). Face validity was considered acceptable. EFA showed a factor structure similar to the original EDI. Correlations (range: 0.32 to 0.67) and agreements between GEDI scores and other German language instruments suggested good external reliability. Scoring within the lowest 10th percentile was strongly associated with age.

**Conclusions:**

Our psychometric assessment suggests good reliability and consistency of the GEDI. Differences in the age distribution of children, pedagogical objectives and educational system features of German preschools require future work to determine score thresholds indicative of vulnerability. Aside from dropping selected items from the original EDI that were inconsistent with features of the German educational system, the distribution of values in the language and cognitive development domain also suggested that context-specific cut-offs must be established for the German version. Such efforts are needed to account for relevant contextual differences between the educational systems.

## Background

Early childhood health and development sets the foundation for health and well-being in later life [1–3]. Therefore, public health should ensure the healthy development of all children. However, due to differences in biological factors and environmental conditions, not all children develop at the same rate or in the same sequence. Hence, it is important to be able to detect relevant physical, socioemotional, or cognitive delays, and to differentiate between “real delays” and “developing in an slightly alternative chronology” starting no later than age three, when intervention may be most effective [4, 5].

In Germany, population-level measures to detect children at developmental risk and to enable early supportive interventions are limited. First, a required annual school entry health examination is performed on all children planning to enter school. However, this examination includes the administration of few, if any, standardized tests such as the Stengths and Difficulties Questionnaire (SDQ) [6] or the Social-Paediatric Developmental Screening for School Entry Health Examinations (Sozialpädiatrisches Entwicklungsscreening für Schuleingangsuntersuchungen – [SOPESS]) [7]. Additionally, the timing of this examination at or after age four reduces opportunities for early intervention and fails to account for continually evolving social and emotional competencies. Second, a variety of non-validated measurements are routinely used for documenting child development in German preschools, a setting with a 90% attendance rate for children between the ages of 3 and 6 years [8]. The choice of instrument often depends on the preferences of preschool directors or those of preschool organizations, complicating efforts to generate a standardized assessment of child development on a population level [9, 10]. Moreover, the few validated instruments currently available (e.g., SDQ [11], Dortmunder Entwicklungsscreening für den Kindergarten 3–6 R [DESK] - *Dortmund developmental screening for preschool* [12]) have limited utility as population-based tools for detecting at-risk children. For example, some fail to assess key developmental domains while others are relatively lengthy and less efficient as they cover secondary developmental domains (e.g., music and arts) or include time-consuming tasks that may place a considerable time burden on those performing the assessment.

Preschool is an ideal setting for assessing early child development. Besides providing substantial access to the target population, teachers in preschools have close daily contact with children similar to that of parents and are thus well positioned to assess their development reliably [13, 14]. In addition, given that many preschools in Germany are organized at the municipal level, a preschool-based approach could offer valuable public health guidance for communities.

In the international literature, several instruments to measure development quantitatively in the preschool setting have been reported [15–23]. One of the most well-established instruments that allows population-level data aggregation and can be used as a community-level surveillance instrument is the Early Development Instrument (EDI) [24–26]. The psychometric characteristics of this survey-based tool have been demonstrated in its country of origin [27] and in a variety of international settings [24–26, 28]. Until recently, a German language version of the EDI has not been available. Therefore, the objective of this study was to evaluate the psychometric properties and to analyze the contextual appropriateness of a new German version of the EDI (GEDI) for use in German preschool settings.

## Methods

### Setting and subjects

We tested the psychometric properties and contextual appropriateness of the GEDI in southwest Germany. As preschools in Germany are administered through a variety of mechanisms including private, community-, local government- and faith-based organizations, we recruited schools by contacting the individual responsible at each organization. Recruitment took place from December 2015 to June 2016, with invitations extended by email or telephone to 27 preschool organizations throughout the northern portion of the federal state of Baden-Württemberg (population ca. 11 million). Ultimately, six organizations took part in our study. Of these, three were located in a small city (population ca. 30,000) participating in a larger project to promote health and well-being [29] (http://www.ein-gutes-jahr-mehr.de), and three were located elsewhere in two additional cities. A total of nine preschools belonging to these organizations with 444 children and 60 teachers were available for participation in the study. Inclusion criteria for participating teachers in each preschools were having established a relationship with an eligible child for at least 1 month, having sufficient German language knowledge, and having taken part in a training session prior to the assessment. Eligibility criteria for the children to whom the GEDI was administered included age 3 to 6 years, absence of special needs and parental consent. Sixty teachers from nine preschools completed the GEDI for 225 children (51% of eligible children). Our reporting is based on an extension of the STROBE Statement [30].

### Assessment of early childhood development using the EDI

The EDI consists of 103 items (administration time: 10–20 min) and provides detailed information on five key developmental domains: Physical Health & Well-being (PHY) (13 items), Social Competence (SOC) (26 items), Emotional Maturity (EMO) (30 items), Language and Cognitive Development (LAN) (25 items) and Communication and General Knowledge (COM) (8 items). Twenty-six supplemental items assess information on the preschool, on sociodemographic characteristics of the child (e.g., immigration status, primary language), past health or special needs, and on the type of care arrangement before entering preschool (e.g., previous enrollment in a nursery, use of an *au pair*). To maximize standardization in administering the GEDI in preschools, all participating teachers (N = 60) underwent in-person training on the content, aims and use of the instrument, similar to procedures used in the development of the original EDI.

The items of the original EDI [31] are rated using 2-point yes/no questions or 3-point scales (often/very true, sometimes/somewhat true, and never/not true; very good/good, average, poor/very poor). Following procedures outlined in the original report [31], we recoded all items for the GEDI-validation data set on a scale of 0 to 10. Mean GEDI scores were calculated for each of the five domains, with higher scores indicating better development. Domain scores were excluded from analyses if a child had three or more missing values for items within a given domain [31]. We did not exclude complete cases because of missing data and followed the same approach to desctibing our sample as in the original EDI calidation paper. In the absence of a normative German sample to establish valid cut-offs, and in line with the original EDI procedures, children who scored lower than the 10th precentile in at least one of the five domains were preliminarily categorized as “vulnerable” in terms of school readiness [32]. However, we were aware that German children scoring below the 10th percentile cut-off might not be vulnerable *per se*, given the differences between the Canadian and the German educational systems and examined this possibility in an analysis described below.

### Instrument translation process

With the permission of the EDI authors, the GEDI was created through a translation process by the research team and English native speakers consulted for the study, with back translation conducted by a second, independent native English-speaking expert linguist [33]. Differences between the original and back translation versions were discussed until consensus was reached between the translators, back translators and members of the original Canadian EDI research team.

### Instrument modification

To provide an accurate and meaningful translation, it was necessary to replace three of the 26 supplemental items due to a lack of applicability in the German context. These included assessment of class type, aboriginal/indigenous status and ethnicity. These items were replaced with items to assess group structure, immigration status and country of origin. As the organizational structure and pedagogical objectives of German preschools vary significantly, we included four additional items potentially associated with early childhood development: overall educational goals of the preschool, German as a second language, the availability of additional educational resources (i.e., language skills, art and music instruction, physical activity), and categories for the length of the daily stay at the preschool (up to 5 hours, fige to 7 hours, greater than 7 hours). These characteristics were not reported in the results of the current study, but are rather mentioned for the sake of completeness. Four indicators of socioeconomic status (SES) included in the original EDI (i.e., family income/wealth indicator, parental education, emploment and siblings) were moved to a separate parental survey used to more extensively assess sociodemographic factors as well as the health and family background of the children.

### Assessment of sociodemographic factors

The measurement was adapted from the standard socioeconomic Index [34] (see Table 5) consisting of three components: family income, maternal education, and parental employment. Consistent with federal standards for reporting poverty and wealth and the recommendations for reporting on social cohesion in Europe, household income was determined according to need [34–36].

### Psychometric evaluation

Reliability was assessed through checks of internal item consistency, test-retest response and interrater reliability. Internal consistency of items within each of the five domain scales of the GEDI was tested using Cronbach’s alpha. Domain intercorrelations were assessed using Pearson correlation coefficients. To establish test-retest reliability, preschool teachers were asked to complete the GEDI for a second time for a subset of randomly selected children (*n* = 29) after a two-week interval. To establish interrater reliability, preschool teachers were instructed on how to randomly select a subset of children (*n* = 27) and the GEDI was completed after an interval of 2 weeks by a different teacher also acquainted with the child for at least 1 month. For both assessments, we confirmed that the child’s data from the first and second measurement time and from both assessors agreed by comparing the following unchanging demographic variables: date of birth, gender, and special needs status.

The validity of the GEDI was explored in several ways: 1) Content validity was assessed using a face-validity approach through qualitative interviews with preschool teachers. 2) Concurrent validity was assessed by comparing Pearson correlation coefficients and by plotting differences using Bland-Altman plots [37] between the mean GEDI scores and those of other previously validated instruments including the SDQ [11] and the DESK 3–6 R [38, 39], administered concurrently (see below). 3) Convergent validity was assessed using exploratory factor analyses (EFA). 4) External validity was assessed through comparison of correlations between GEDI scores and sociodemographic parameters of our sample with those of previous studies using the EDI.

Face-validity was determined following consultation with three preschool teachers, one of whom participated in our study and two who worked at non-participating preschools. Each rated the GEDI in five areas on a 5-point ordinal scale ranging from *very bad/low* to *very good/high* regarding the comprehensibility of items, adequacy of examples provided in the items, adequacy of item coverage for key developmental domains, balance of effort and information utility, and usefulness in day-to-day work.

To assess concurrent validity, we collected data from the same individuals using two existing instruments currently applied in some German preschools: the SDQ and the DESK 3–6 R.

#### SDQ

The SDQ is a brief, internationally standardized instrument for screening at the individual level consisting of 25 items assessing social skills and emotional maturity in five domains: emotional symptoms, conduct problems, hyperactivity/inattention, peer relationship problems, prosocial behavior. It has been widely used in both clinical and community settings throughout the world [11, 40–42]. The SDQ was completed by preschool teachers for all participating children. As the SDQ domains are conceptually close to the EDI domains “social competence” and “emotional maturity”, we expected significant associations and agreement between corresponding GEDI and SDQ domains.

####  DESK 3–6

The DESK was developed in Germany for monitoring children’s individual developmental behavior in preschool during their daily routine, including direct performance tasks [39]. It covers developmental domains comparable to the GEDI, but is available in age-specific questionnaires and includes group performance tasks that result in administration time of at least 40 min per child. Given this feature, teachers in our study were instructed in selecting a random subsample of six to nine children per preschool depending on time availability (desired n of completed assessments = 72). We expected significant associations and acceptable agreement between corresponding GEDI and DESK domains, despite methodological differences between the two instruments.

We interpreted the size of a correlation coefficient according to Hinkle et al. [43] (0.0 to 0.3 negligible; 0.3 to 0.5 low; 0.5 to 0.7 moderate; 0.7 to 0.9 high; 0.9 to 1.00 very high).

We used EFA to evaluate convergent validity. As the main domain structure of the original EDI [31] mirrors what is known from early developmental psychology [44, 45], we chose to define these five domains as given latent factors and examined the structures of the subfactors within these. Moreover, a promax rotation was performed to assess correlation in the extracted factors. To define the number of subfactors within the main domains, the Kaiser criterion (eigenvalues ≥1.0) was used.

For assessing potential external validity, we determined the distributions of the GEDI scores in our sample using kernel density plots and compared 10th percentile values with figures reported in the original EDI validation study. Moreover, we conducted logistic regressions assessing domain scores and odds ratios of the German sample for scoring in the lowest 10th percentile with regard to SES, German as second language, immigrant status, and sex to be able to draw conclusions on the similarity of our figures compared to those from other countries.

### Data management and analysis

To ensure data quality, a 10% random sample of data was selected by the research team and re-entered by a commercial data entry service, resulting in an error rate of 0%. Additionally, congruence of responses given by teachers and parents was confirmed for the child’s date of birth, gender, country of origin, and first language.

To meet the requirement for normality in creating Bland-Altman plots and to enable cross-measure comparisons, we transformed scores for the GEDI (sub-)domains and the corresponding SDQ−/DESK-domains (the outcome variables) into z-scores. To determine whether additional transformation of the scores was necessary, we assessed the distribution of differences between the measures using ladder-of-powers histograms [46, 47]. Bland-Altman plots were then generated using the Stata “-concord” command for all comparisons [48]. Each graph plots the mean of the measures against the difference between the measures. In each plot, the middle horizontal line represents the mean difference between the measures while the outer lines indicate 95% confidence intervals for agreement [49]. The association between each of the GEDI comparisons with SDQ and DESK was examined (i) by considering the mean difference and (ii) the scattering of dots around this line in relation to the latent trait continuum on the x-axis. Bland-Altman plots, generated to enable subgroup analyses by age group (3 years, 4 years, 5 to 6 years), are presented in the appendix (Additional file 1).

All statistical analyses were conducted using the statistical software package STATA (Ver. 13.1 for Mac, StataCorp LP, College Station, TX).

## Results

### Recruitment

**Fig. 1:**
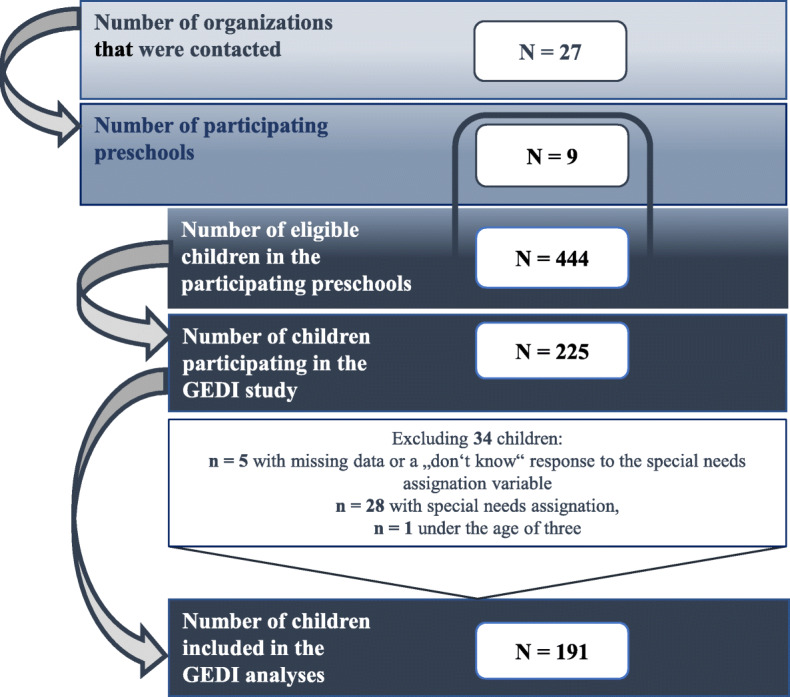
Recruitment Process – Flow Chart

The recruitment process is presented in Fig. 1. Reasons for lack of participation in some preschool organizations included a shortage of staff or the subjective perception of the EDI as a deficit-oriented instrument. Some preschool teachers also noted that involvement in the development of a population-based instrument for primary use in research and policymaking was not a strong motivator for participation. Indeed, some individuals felt the need for an instrument to assess individual children was more important. Due to time constraints, participation in retest- and interrater reliability testing was limited to four preschools and completion of the validated DESK survey for assessment of concurrent validity took place in seven of the nine recruited preschools.

### Sample description

All children to whom the GEDI was administered (*N* = 225) were additionally assessed with the SDQ. For several reasons, 34 children were excluded from analyses (see Fig. 1) leaving 191 children (85%) in the analytic sample. The DESK was administered to a subgroup of 39 children (17%) (age three: *n* = 14, age four: *n* = 15, age five: *n* = 7, age six: *n* = 3) in seven preschools.

The average age of children in the analytic sample was 4.72 years (SD 1.05; range: 3 years to 6 years, 9 months; age three (*n* = 58; 30.4%), age four (*n* = 60; 31.4%), age five (*n* = 43; 2.5%), age six (*n* = 30; 15.7%)). Forty-nine percent of the analytic sample was female, and 2.6, 49.2 and 40.3% had a low, middle or high SES, respectively. Eighteen percent spoke German as a second language.

### Reliability of the GEDI

Descriptive statistics (means, standard deviations, and internal consistency coefficients) for the five domains of the GEDI are presented in Table 1, along with the descriptive statistics reported in parentheses for the original sample used in the development of the EDI [50]. Cronbach’s alphas for each domain of the GEDI were generally very good (> 0.8). Only the domain PHY showed lower, but acceptable internal consistency (0.69).

**Table 1:** Comparison of mean GEDI domain scores with those from the EDI Normative Sample

Domain (number of items)	N	M (***Normative Sample***^**a**^***)***	SD ***(Normative Sample***^**a**^***)***	α ***(Normative Sample***^**a**^***)***
Physical Health and Well-Being (13)	189	8.78 *(8.74)*	1.29 *(1.11)*	0.69 *(0.84)*
Social Competence (26)	191	8.17 *(8.20)*	1.49 *(1.82)*	0.91 *(0.96)*
Emotional Maturity (30)	188	7.54 *(7.99)*	1.40 *(1.56)*	0.89 *(0.90)*
Language and Cognitive Development (25)	184	5.31 *(8.28)*	2.38 *(1.91)*	0.93 *(0.93)*
Communication and General Knowledge (8)	191	8.06 *(7.65)*	2.17 *(2.04)*	0.88 *(0.94)*

* GEDI* German version of the Early Development Instrument

^a^Descriptive statistics drawn from the “gold standard” normative sample (*N* >  124.000 <  125.218) of the EDI (from Janus and Duku 2004: Normative Data for the Early Development Instrument). Data courtesy of the Mannheim Institute of Public Health

Test-retest reliability analyses from 18 children and interrater reliability analyses from 19 children did not yield statistically significant differences between the first and second measurements nor between teacher one and two for any of the GEDI domains. Pearson correlations suggested moderate to high test-retest reliability ranging from 0.50 to 0.81 (*p* < 0.05) and interrater reliability ranging from 0.48 to 0.71 (*p* < 0.05).

### Validity of the GEDI

#### Content validity

Preschool teachers rated the comprehensibility of items on average as 4.0 (SD 1.65), adequacy of examples provided in the items on average as 4.3 (SD 1.81), and coverage of all relevant developmental domains as 4.0 (SD 1.65), each on a 5-point ordinal scale.

#### Concurrent validity

Table 2 highlights associations between SDQ and DESK scores and corresponding GEDI scores. In general, SDQ domains demonstrated small negative correlations with the two GEDI domains SOC and EMO (− 0.32 and − 0.47; *p-values* < 0.001), indicating a positive association in the construct, due to the wording of the SDQ items. Moreover, we found small to moderate positive correlations between corresponding DESK and GEDI domains (0.35–0.67; *p-values* < 0.05).

**Table 2:** Concurrent validity: Correlations between GEDI, SDQ and DESK domain scores

GEDI	PHY	SOC	EMO	LAN	COM
**SDQ (*n*** **= 184 to 191)**					
Emotional symptoms	− 0.43***	− 0.25***	− 0.37***	− 0.11	− 0.28***
Conduct problems	− 0.17**	− 0.32***	− 0.34***	− 0.14*	− 0.15*
Hyperactivity/inattention	−0.28***	− 0.43***	− 0.47***	− 0.17*	−0.23***
Peer relationship problems	−0.34***	−0.35***	− 0.35***	−0.06	− 0.25***
Prosocial behavior	0.14*	0.13	0.05	0.12	0.1
**DESK (*n*** **= 39)**					
FMO^a,b,c^	0.38*	0.27	0.3	0.29	0.28
GMO^a,b,c^	0.35*	0.43**	0.32	0.36*	0.28
SZK^c^	−0.15	−0.31	−0.59	0.11	−0.02
SZV^a,b^	0.25	**0.39**	**0.34**	0.18	0.47*
SZI^c^	−0.44	−0.50	−0.50	0.05	−0.44
AKN^c^	−0.06	0.23	−0.32	−0.46	0.21
KSP^a^	0.5	0.5	0.15	0.24	**0.55**
KOG^b^	0.12	0.40	0.14	0.67*	0.38
BKS^c^	0.00	0.14	−0.79**	0.05	0.18
BKM^c^	−0.08	0.04	−0.71*	−0.02	− 0.22
SPK^b,c^	0.02	0.34	0.18	0.37	0.47*

*GEDI* German version of the Early Development Instrument, *SDQ* Strengths and Difficulties Questionnaire, *DESK Dortmund developmental screening for preschool 3 to 6 years*, *PHY* physical health and well-being, *SOC* social competence, *EMO* emotional maturity, *LAN* language and cognitive development, *COM* communication and general knowledge, *FMO* fine motor skills, *GMO* gross motor skills, *SZK* social competence, *SZV* social behavior, *SZI* social interaction, *AKN* attention and concentration, *KSP* cognition and language, *KOG* cognition, *BKS* basic competence literacy, *BKM* basic competence numeracy, *SPK* language and communication

Numbers **in bold** indicate the correlations between corresponding GEDI and DESK domains

^a^3-year-olds (*n* = 14), ^b^4-year-olds (*n* = 15), ^c^5- to 6-year-olds (*n* = 10)

* *p* < 0.05; ** *p* < 0.01; *** *p* < 0.001

### Agreement between methods

The Bland-Altman method requires normally distributed differences between measures. Ladder-of-power histograms for differences between variable pairs showed approximate normality, indicating that further transformation was not needed. We created plots for domain pairs, in which we expected to observe agreement with each other and with regard to the latent construct. Moreover, we assessed mean differences in plots by different age groups (3, 4, and 5 to 6 years of age) and for the overall sample (Table 3). The overall bias (mean difference) was close to zero for most comparisons except for domain pairs FMO/PHY_3 in three- and five- to six-year-olds, in GMO/PHY_3 in four- and five- to six-year-olds, and in SZK/EMO _1 and AKN/EMO_4 in four- and five- to six-year olds. The table also shows that mean differences for GEDI/SDQ domain pairs predominantly range below zero in younger children (three- and four-year-olds) and above zero in five- and six-year-old children in our sample. For the GEDI-DESK-domain pairs, we could not observe a consistent pattern in the mean differences. Age-specific plots are provided in the appendix (Additional file 1).

**Table 3:** Concurrent validity: Mean differences between selected GEDI and SDQ/DESK domain pairs^a^

	Mean difference [95% limits of agreement]			
**Domain pairs**	**Age groups**			
**SDQ/GEDI**	**3** ***n*=58**	**4** ***n*=60**	**5 & 6** ***n*=73**	**overall** ***N*=188-191**
peers/SOC_1	-0.13 [-2.32 to 2.06]	-0.16 [-1.87 to 1.55]	0.24 [-1.57 to 2.05]	0.003 [-1.92 to 1.93]
peers/EMO_1	-0.33 [-2.91 to 2.24]	-0.24 [-2.73 to 2.26]	0.52 [-1.45 to 2.48]	0.017 [-2.43 to 2.46]
prosocial/EMO_1	-0.24 [-2.92 to 2.44]	-0.31 [-2.82 to 2.21]	0.50 [-2.29 to 3.29]	0.019 [-2.74 to 2.78]
conduct/EMO_3	0.07 [-1.65 to 1.8]	-0.12 [-2.46 to 2.23]	0.03 [-1.70 to 1.76]	-0.003 [-1.94 to 1.94]
hyper/EMO_4	-0.08 [-1.81 to 1.65]	-0.10 [-1.99 to 1.78]	0.15 [-1.52 to 1.82]	0.003 [-1.76 to 1.77]
**DESK/GEDI**	**3** ***n*=14**	**4** ***n*=12-15**	**5 & 6** ***n*=10**	**overall** ***N*=13-39**
FMO/PHY_3	-0.49 [-2.58 to 1.59]	0.03 [-1.81 to 1.87]	0.66 [-1.46 to 2.79]	0.004 [-2.14 to 2.15]
GMO/PHY_3	0.17 [-2.11 to 2.45]	-0.47 [-2.52 to 1.58]	0.48 [-1.48 to 2.45]	0.004 [-2.20 to 2.21]
SZV/SOC_1	-0.12 [-2.05 to 1.81]	0.29 [-1.95 to 2.52]	-	0.067 [-2.01 to 2.14]
KOG/LAN_1	-	-0.36 [-2.35 to 1.63]	-	-0.398 [-2.33 to 1.53]
SPK/COM	-	-0.06 [-2.08 to 1.97]	0.38 [-1.58 to 2.34]	0.121 [-1.84 to 2.09]

*GEDI* German version of the Early Development Instrument, *PHY_3* gross & fine motor skills, *SOC_1* overall social competence with peers, *EMO_1* prosocial and helping behavior, *EMO_3* aggressive behavior, *EMO_4* hyperactive and inattentive behavior, *LAN_1* basic literacy, *COM* communication and general knowledge, *SDQ* Strengths and Difficulties Questionnaire, *peers* peer relationship problems, *prosocial* prosocial behavior, *conduct* conduct problems, *hyper* hyperactivity/inattention, *DESK* Dortmund developmental screening for preschool, *FMO* fine motor skills, *GMO* gross motor skills, *SZK* social competence, *SZV* social behavior, *SZI* social interaction, *AKN* attention and concentration, *KOG* cognition, *SPK* language and communication, - no observations for the age group in this DESK domain, *N.B.* Mean differences for comparisons with fewer than five observations are not included

^a^Mean differences were standardized using z-score transformation

**Fig. 2:**
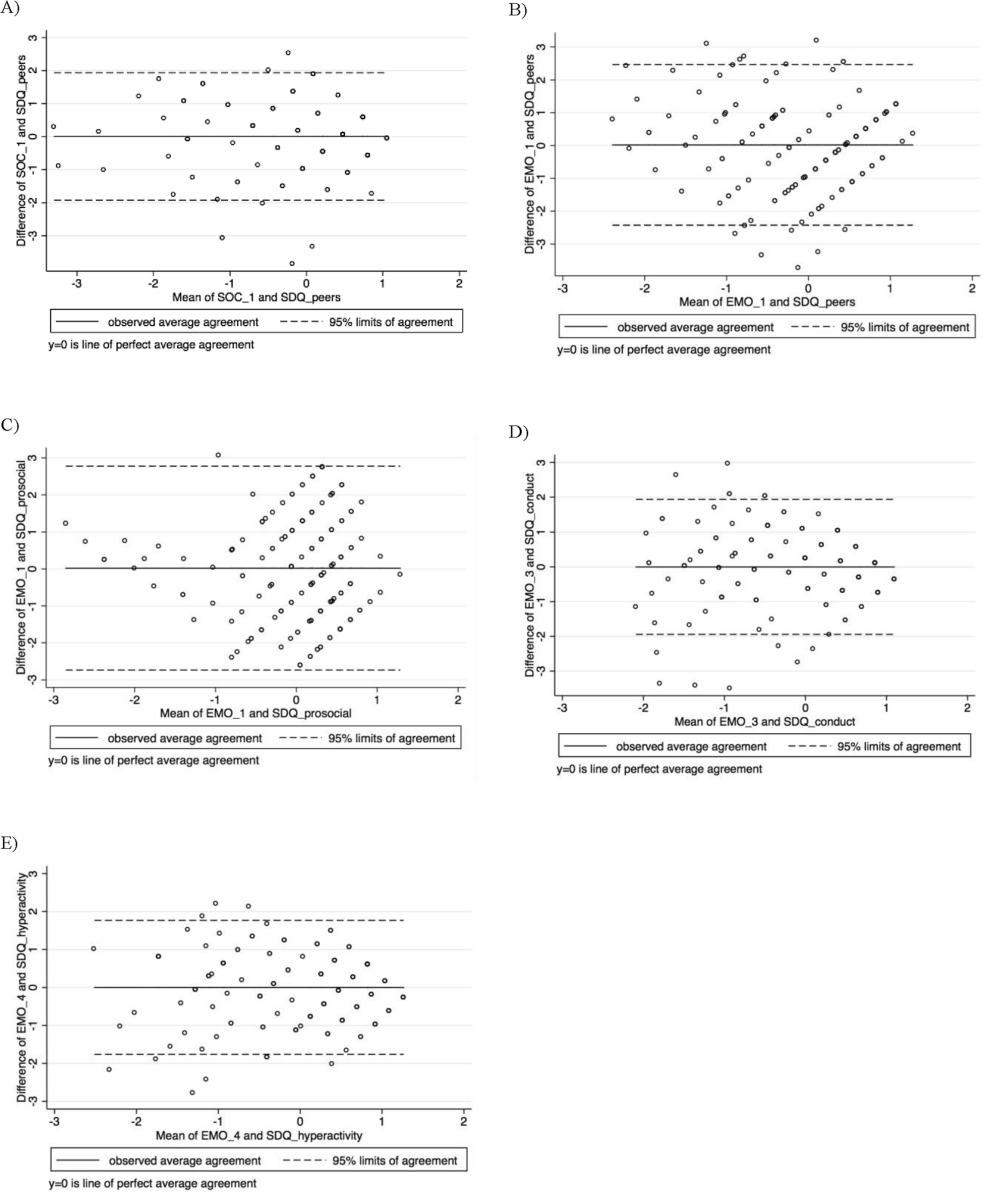
Bland-Altman plots showing agreement between GEDI and corresponding SDQ domain score pairs. The metric for both x- and y-axes in each graph is the z-score for mean domain scores and the difference between scores, respectively

**Plots A to E in** Fig. 2 show dispersion in the extent of agreement between GEDI and SDQ domain score pairs. Generally, we observed good and acceptable agreement in all plots, particularly at the highest end of the latent trait continuum, as points were more tightly clustered around the mean difference line. In the midsection of plots A, B and C and in the lower section of plots D and E, we noted greater dispersion indicating poorer agreement for children with average and lower abilities, respectively.

**Fig. 3:**
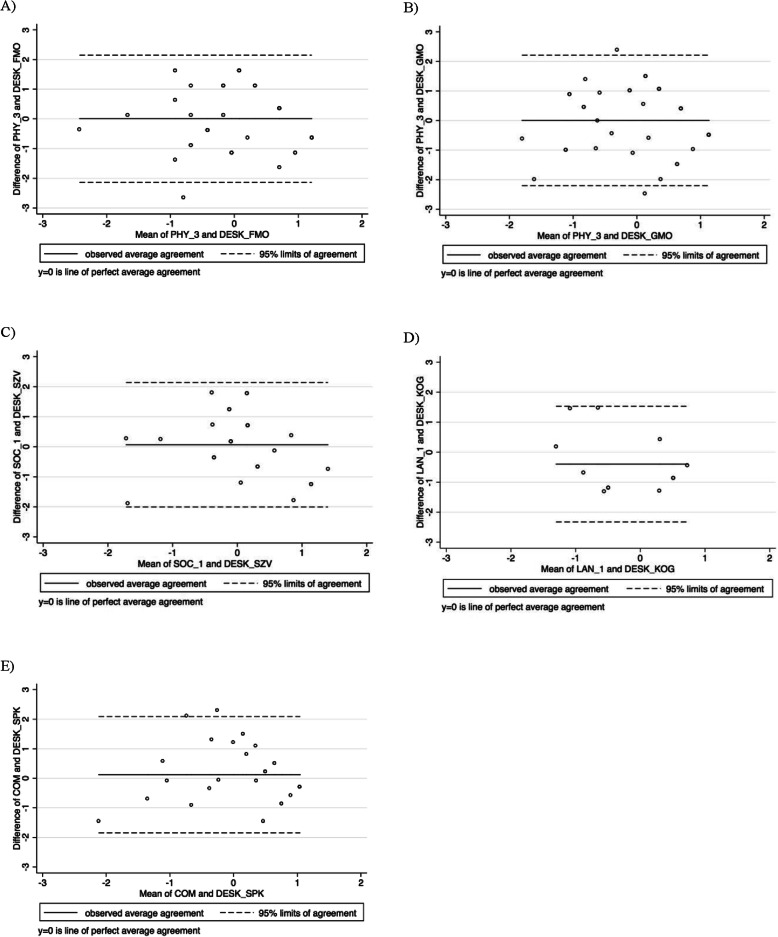
Bland-Altman showing agreement between GEDI and corresponding DESK domain score pairs. The metric for both x- and y-axes in each graph is the z-score for mean domain scores and the difference between scores, respectively

**Plots A to E in** Fig. 3 demonstrate dispersion in the extent of agreement between GEDI and DESK domain score pairs. Aside from a few outliers, we observed generally good and acceptable agreement in all plots, particularly at the highest end of the latent trait continuum, where points were more tightly clustered around the mean difference line. We noted strong ageement at the lowest end of plot A. In the midsection of plot B, we observed greater dispersion indicating poorer agreement for children with average abilities.

### Convergent validity

Several differences were noted when comparing results from the EFA of the GEDI with those from the original EDI report [51] (Table 4): The GEDI, for example, had smaller factor loadings in the main domains PHY, SOC, and EMO, and larger loadings in the main domain LAN and COM. Nevertheless, in all but two of the main domains (PHY and LAN), we found similar factor and subdomain headings. In the main domains PHY and SOC, some items with very small loadings were retained as their content was considered to be strongly related to either physical health or social competence (*hungry, is independent in washroom habits most of the time, shows an established hand preference, is able to solve day-to-day problems by him/herself, is able to follow one-step instructions).* In contrast, because the item *sucks thumb/finger* in the main domain PHY had a very small factor loading and its content was considered more closely related to the domain EMO [52], it was excluded. Additionally, a factor analysis of the domain PHY excluding this item resulted in a two factor model explaining a higher proportion of the variance compared with the model including the item. Therefore, our analysis suggested the presence of 15 rather than 16 factors (subdomains) across the main domains. We elected to alter the subdomain headings for the domain LAN from the one used in the original report of the EDI, as we felt the items underlying the factors in our EFA did not refere to language and cognitive abilities per se, but rather to the availability of resources in German preschools to facilitate development of those abilities.

**Table 4:** Summary of the exploratory factor analyses

Domain	Factor	EDI^**a**^	GEDI
		***subdomain label***	***subdomain label***
		N of items	N of items
		(range)	(range)
		internal consistency (⍺)	internal consistency (⍺)
**PHY**		***physical readiness for school work***	***physical well-being and readiness for school work***
	1	4	6 (*2 items < 0.3*)
		(0.640–0.773)	(0.206–0.628)
		0.715	0.565
		***physical independence***	
	2	4	
		(0.401–0.657)	
		0.256	
		***gross & fine motorskills***	***physical development and gross & fine motor skills***
	3	5	6 (*1 item < 0.3*)
		(0.763–0.897)	(0.251–0.797)
		0.918	0.759
**N item total**		**13**	**12**
**SOC**		***overall social competence with peers***	***overall social competence with peers***
	4	5	5 (*1 item < 0.3*)
		(0.576–0.754)	(0.235–0.796)
		0.862	0.754
		***respect and responsibility***	***respect and responsibility***
	5	8	8
		(0.623–0.823)	(0.394–0.786)
		0.921	0.813
		***independence and adjustment***	***independence and adjustment***
	6	9	9 (*1 item < 0.3*)
		(0.504–0.778)	(0.295–0.831)
		0.911	0.854
		***readiness to explore new things***	***readiness to explore new things***
	7	4	4
		(0.677–0.892)	(0.591–0.857)
		0.863	0.758
**N item total**		**26**	**26**
**EMO**		***prosocial and helping behavior***	***prosocial and helping behavior***
	8	8	9
		(0.777–0.881)	(0.354–0.843)
		0.944	0.88
		***anxious and fearful behavior***	***anxious and fearful behavior***
	9	8	7
		(0.513–0.822)	(0.402–0.804)
		0.808	0.784
		***aggressive behavior***	***aggressive behavior***
	10	7	8
		(0.542–0.795)	(0.401–0.737)
		0.862	0.836
		***hyperactive and inattentive behavior***	***hyperactive and inattentive behavior***
	11	7	6
		(0.559–0.833)	(0.430–0.789)
		0.921	0.834
**N item total**		**30**	**30**
**LAN**		***basic literacy***	***basic literacy and numeracy / resources to learn those abilities are provided in German preschools***
	12	8	9
		(0.043–0.477)	(0.495–0.886)
		0.751	0.899
		***interest and memory***	***interest and curiosity in basic literacy and numeracy***
	13	5	8
		(0.383–0.757)	(0.404–0.786)
		0.779	0.834
		***complex literacy skills***	***cognitive abilities***
	14	6	6
		(0.541–0.766)	(0.397–0.728)
		0.808	0.735
		***basic literacy and numeracy***	***complex literacy skills / resources to learn those abilities are not provided in German preschools***
	15	7	3
		(0.409–0.749)	(0.706–1.004)
		0.802	0.836
**N item total**		**26**	**26**
**COM**		***communication skills***	***communication skills***
	16	8	8
		(0.279–0.924)	(0.525–0.829)
		0.931	0.877

*PHY* physical health and well-being, *SOC* social competence, *EMO* emotional maturity, *LAN* language and cognitive development, *COM* communication and general knowledge

^a^Janus et al. 2005: Early Development Instrument: Factor structure, sub-domains and multiple challenge index

### Distribution of our sample

**Fig. 4:**
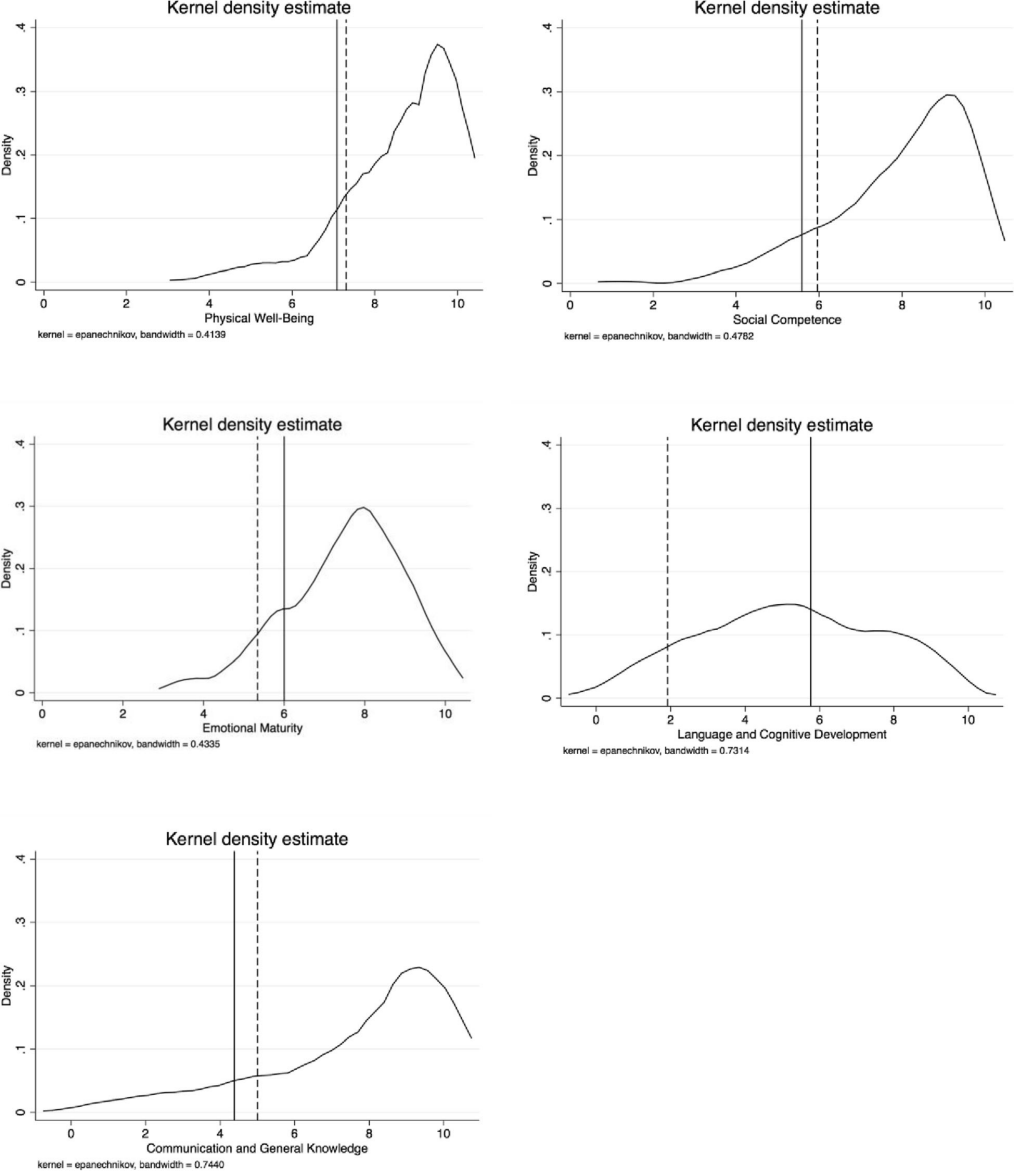
Density plots for all GEDI main domains “density” specifies the kernel function for use in calculating the kernel density estimate. The default kernel is the Epanechnikov kern; the broken line represents the cut-off of the lowest 10th percentile calculated using the study sample of the GEDI; the continous line represents the cut-off of the lowest 10th percentile from the report of the sample used to develop the EDI

The kernel density plots (Fig. 4) for each domain illustrate the underlying distribution of the data. In the domains PHY, SOC, and COM, the majority of children (> 50%) scored in the upper range of the latent trait continuum (> 8.5). With regard to EMO, more than 50% of children scored above 7.6 and children in the top 10th percentile scored above 9.0. Only the domain LAN did not show a similar distribution, with 50% of children scoring in the lower half of the latent trait continuum (≤5).

Regarding the cut-off of the lowest 10th percentile, we found similarities between the German and original sample cut-offs in all domains except LAN (Fig. 4).

#### Sociodemographic characteristics

Table 5 shows that girls in our sample were less likely to score within the lowest 10th percentile compared to boys (odds ratio [OR] 0.74, *p*-value > 0.05). It also indicates that children who learned German as a second language were more likely to score within the lowest 10th percentile compared with children who were native German speakers (OR 3.22, *p*-value < 0.05). Independent from gender, younger children were more likely to score within the lowest 10th percentile (OR for three-year-olds compared to six-year-olds: 3.22, *p*-value < 0.05). Finally, although the number of children from families with low SES was small, they did not have significantly greater odds of scoring within the lowest 10th percentile compared with children from families with a high SES.

**Table 5:** External validity: GEDI domain scores and odds ratios^x^ by selected characteristics

	n/N (%)^**b)**^	aOR^**#**^ [95% CI]
**German as a second language**		
yes	15/34 (44.1)	3.22^a)^ [1.38, 7.50]
no	41/157 (26.1)	1
**n within lowest 10th percentile / N full sample (%)**	**56 / 191 (29.3)**	
**Immigrant status**		
yes	12/30 (40)	2.59^a)^ [1.06, 6.34]
no	43/159 (27.0)	1
**n within lowest 10th percentile / N full sample (%)**	55 / 189** (28.8)	
**Age**		
3 years	24/58 (41.4)	3.22^a)^ [1.07, 9.71]
4 years	21/60 (35)	2.71 [.90, 8.19]
5 years	6/43 (13.95)	0.77 [.21, 2.83]
6 years	5/30 (16.67)	1
**n within lowest 10th percentile / N full sample (%)**	**56 / 191 (29.3)**	
**Sex**		
male	98/191	1
female	93/191	0.74^#^[0.37, 1.48]
**n within lowest 10th percentile / N full sample (%)**	**56 / 191 (29.3)**	
**SES-Index**^**1**^		
low	2/5 (40.0)	2.60 [.32, 21.34]
middle	28/94 (29.8)	1.28 [.63, 2.57]
high	20/77 (26.0)	1
**n within lowest 10th percentile / N full sample (%)**	50 / 176* (28.4)	

^x^for preschool children scoring in the lowest 10th percentile in at least one of the five domains

^1^SES-Index: Components included net family income (dividing the sum of net income through the sum of the family’s members’ weighting), maternal education, and parental employment, each component can take values up to seven, and the total SES-Index ranges between 3 and 21 and is categorized as follows: low: 3–8, medium: 9–14, high: 15–21

*16 of 191 children were missing data from the parental survey

** Two children provided no data for this variable

a) *p* < 0.05; b) using 10%-cut-offs calculated using the study sample of the GEDI; # adjusted for age

## Discussion

### Summary of main results

In this study, we were able to demonstrate good reliability and confirmed several aspects of validity for the GEDI.

Specifically, we observed excellent internal consistency and moderate to good test-retest and interrater reliability. Moreover, we confirmed face validity by conducting expert interviews with preschool teachers. Additionally, our findings suggest that most items included in the GEDI are valid indicators for key developmental domains. Testing the GEDI against validated instruments currently in use in Germany showed good correlations between corresponding domains. Bland-Altman plots overall revealed good and acceptable agreement between GEDI and SDQ/DESK-measured domains of child development, respectively. However, some variation in agreement existed across the distribution of scores and between age groups, with the GEDI underestimating scores in younger age groups and overestimating in older age groups. In addition, associations between GEDI scores and specific characteristics such as SES and sex were comparable to previous work [24, 26, 28, 32, 53], suggesting good convergent validity. Lastly, our density plots displayed left-skewed distributions of GEDI scores across domains, as might be expected. Scores in the lowest 10th percentile were largely similar between the German and the original EDI in all domains except LAN.

### Reliability of items

Although internal consistency was generally good, some exceptions indicate a need for careful consideration. Two domains in particular should be given a closer look: PHY and LAN.

In terms of content, it might be that the domain PHYcontains more than a single latent variable; however, the original item structure of the EDI, which we used as a template, treated this as one domain. Within this domain, four items loaded below 0.3.: (i) the loading for S*ucks thumb/finger* was near zero and the item-total correlation was quite low (α = 0.077). Similarly, the original EDI report [51] as well as a report from Hagquist et al. (2013) showed poor loading (0.401) of this item in factor analyses and identified it as the most poorly fitting item in the domain [54]. Excluding this item from our analyses, however, did not result in significantly higher internal consistency as the item response category used by 92% of our sample was “never or not true”. One potential explanation for the latter finding is that the vast majority of children in our sample came from families with a higher SES, a setting which might provide greater emotional stability [55]. Given that theories of developmental psychology consider this item reflective of emotional conditions in children such as anxiety or depression [52, 56], we recommend shifting the item to the domain EMO. Similarly, three items within the domain PHY showed a lack of variablity in responses: (ii) *child arrives hungry,* (iii) *is independent in washroom habits most of the time*, and (iv) *shows an established hand preference.* We attribute this to selection issues in our study, with the majority of our sample coming from higher SES households. Previous work suggests that children in high-SES families have more of the resources needed to support their positive development than those from lower SES households [57]. Correlations between SES and child development in our study are to be interpreted with caution, however, and future studies implementing the GEDI or an adaptation of it should ensure that children from diverse social backgrounds are sampled.

While many studies show that a secure and organized parent-child attachment is positively associated with the social, emotional and cognitive skills of children [58], only a few items that relate to these skills exist in the GEDI and the EDI as originally reported. Thus, including additional items in the GEDI covering aspects of familial support may, as others have suggested [59], improve the GEDI.

According to our interpretation, the poor performance of three items within the LAN domains across all age groups (*is able to read complex words, is able to read simple sentences, and is able to write simple sentences* [response of “never or not true” in almost all cases [96, 96, 97% respectively]]) might be related to differences in the structure and learning objectives in German versus Canadian preschools. As the context in which the original instrument was developed, Canadian preschools focus from the outset on promoting advanced language and math skills, whereas German preschools emphasize free play during preschool time and introduce children to basic numeracy and literacy skills only in the last year for the oldest children. Advanced reading and writing abilities do not represent pedagogical aims in German preschools, which may explain why children have neither developed nor are expected to have these skills before the first grade in elementary school. A previous study [54] from Sweden, where preschools take a similar approach to that of Germany [60], reports similar results for two of the items mentioned. Moreover, children enrolled in German preschools typically range from 3 to 6 years of age (mean of our sample 4.7), while the age range for children participating in the sample used to develop the original instrument was higher (4 years, 11 months to 6 years, 4 months) [50]. This would suggest, therefore, that preschool children in Germany would be even less likely to show these competencies. Therefore, these items might have to be excluded in order to produce a reliable, contextually appropriate instrument documenting early development and vulnerability in Germany.

### Validity assessment

Our content validity results and a factor structure very similar to that reported in the original study [31] underscore one of the key tenets of developmental psychology - that children’s development is universal [44]. Thus, most of the GEDI items seem to be transferable to children across industrialized countries.

Significant negative correlations between two domains of the SDQ (i.e., SOC and EMO) with comparable domains in the GEDI indicate acceptable concurrent validity. Significant positive correlations of GEDI domains with corresponding DESK domains (all *p*-value < 0.05) indicate good validity in younger children who comprised a majority of the subsample (74%, *n* = 29), whereas older children had very low and in some cases negative correlations in half of the corresponding DESK domains (5/11). We attribute this either to the small sample size of older children, or to the fact that those DESK domains mainly include group performance tasks while GEDI items are based on teacher report [39].

Results from Bland-Altman plots show good to moderate agreement between the GEDI and SDQ/DESK, though with some variation aross the distribution of scores. This was expected, as the measures capture constructs that are similar but not identical. For SDQ, agreement values are derived from the total sample. Acceptable agreement only in the highest scores of GEDI-SDQ domain pairs might be related to scorings in the higher ranges of the latent constructs as shown in the density plots. Furthermore, based on the mean differences, preschool teachers tended to underestimate younger children’s development (age groups 3 and 4 years) and to overestimate older children’s development (age groups five and 6 years) with the GEDI compared to assessments applying the SDQ. This finding suggests that GEDI assessment in Germany should be administered using age-specific questionnaires. In the small subsample in which DESK was administered, we found similar results, except for domain pair GEMO/PHY_3, where the mean difference for three-year-olds was near zero and for four-year-olds was almost half a standard deviation below zero. This inconsistency might be attributed to methodological discrepancies and should be interpreted with caution. Taken together, these findings suggest that further adaptation will be necessary for future use of the GEDI, especially for enabling valid measurement of development in middle and lower score ranges of the latent construct.

Despite a factor structure very similar to the one reported for the EDI [51] (16 instead of 15 factors/subdomains), we observed loadings that were generally lower. This might be due to a smaller sample size of and to a younger mean age in our sample. Therefore, a future study to confirm convergent validity of an adapted GEDI should try to achieve a larger sample and a more uniform age distribution.

### Sample distribution compared to representative data

The density plots reveal distributions as expected in the domains PHY, SOC, EMO and COM. For the domain PHY (also containting items that cover the general state of health and motor abilities of children) our results are in line with national statistics on the general health status of children in this age range [61], in which 57.1, 38.6 and 4.3% report having a very good, good or poor health status, respectively. Another nationally representative study reported, that 5 to 11% of preschool children have noticeable problems in their motor development [62]. This statistic is consistent with findings in our sample, in which a majority of children (> 90%) scored in the upper range of the specific latent trait continuum (right-skewed distribution).

Further, around 17% of children in Germany are affected by psychological health issues [61, 63]. This in consistent with our results from domains SOC and EMO, in which approximately 75% of our sample scored higher than 8.7 and 6.8, respectively.

For COM, only 10% scored below 5 in our sample, a finding consistent with representative monitoring data, in which 3 to 16% of children showed difficulties in the development of communication skills such as vocabulary, speech comprehension, articulation, and oral fluency [64].

For the domain LAN, the picture is different: If we had used the score threshold applied in the report of the original EDI, 50% of the children would have scored below 5 and would have been identified as vulnerable. Report from previous study conducted in Germany, however, suggests that only 20.7% showed deficits in languge and cognitive development [12]. We interpret this as a lack of contextual appropriateness of several items in the domain LAN, as discussed previously.

### Opportunities and barriers of the current version of the GEDI

Our results show that the GEDI was acceptable in the German preschool setting, most items were valid and thus, with further adaptations the GEDI promises to offer a useful tool for monitoring child development at the popoulation level in Germany [31]. In terms of detecting developmentally delayed children, one of the biggest advantages of the GEDI is that it is designed for teacher proxy reports, which makes assessment independent from parental availability related to language barriers or other factors. Moreover, the instrument allows the preschool teacher to reflect on the development of the individual child. If concerns regarding development delay arise from the GEDI assessment, preschool teachers are well-positioned to determine the relevance of the issue for a specific child in question given their own professional capacity.

Cut-off scores to determine vulnerability rates for German preschoolers would have to be generated with a contextually appropriate version of the GEDI adapted to the context in the ways we describe.

In fact, previous studies on the EDI in other countries [24–26, 28] computed vulnerability rates by classifying children in their study population as developmentally vulnerable if they scored in the lowest 10th percentile in at least one domain. In these countries, the age ranges of children in preschools were somewhat narrower (4–6 years), or they assessed only children from one age group, making comparison with the original EDI sample easier.

Taken together, to establish vulnerability cut-off scores that are contextually appropriate for the German system, the adaptation of the GEDI has to account for both (i) the pedagogical objectives of the German preschool context and (ii) age-appropriateness.

### Limitations of our study and future directions

We provide the first evidence of the reliability and validity of a German translation based closely on the internationally renowned EDI instrument. Despite this strength, we acknowledge several limitations to our work. While psychometric evaluations of existing instruments do not require representative samples, for example, a selection bias in our sample makes it difficult to derive reference values and describe child development at the population level. In Germany, a legal requirement exists for active instead of passive consent from children or their parents [65]. While similar selection biases exist in other studies [66, 67], the net result is that participation is greatest among higher SES parents. However, to be useful as a population-based measure, future data should be anonymized and routinely collected in preschools.

While most of our measurements reflected moderate to high reliability, long intervals between the first and second measurement points of our test-retest and interrater reliability check are also a limitation of our study. These were related to the competing time demands of preschool teachers. Nevertheless, correlation values between test and retest were moderate to high, indicating good consistency of data over time. With regard to internal consistency, we followed the recommendations of the COSMIN checklist [68] and were able to show good Cronbach’s alpha values similar to those of the developers [32]. Nevertheless, Cronbach’s alpha has some limitations in assessing the internal consistency of latent variables [69].

While we performed a comprehensive EFA [68], the sample size impeded conducting a confirmatory factor analysis. Moreover, given our small subsample of children assessed with the DESK, we were not able to draw any definitive conclusions on concurrent validity using the DESK. However, using the SDQ as a comparison for the GEDI domains “social competence” and “emotional maturity” in the whole sample exhibited good correlations.

In order to develop an adapted, contextually and age-appropriate version of the GEDI, we suggest the application of Item Response Theory (IRT) [70]. This method has been used for adapting the Swedish [54] and Australian [71] versions of the EDI and may also be suitable for a successful adaptation of the GEDI. We expect IRT analysis to result in a shorter instrument, including only those items with a high information value for age-specific latent trait scopes. This could increase the feasibility of the GEDI for population monitoring in Germany.

## Conclusion

The results of this study give empirical, data-driven guidance towards adapting and refining the GEDI for population monitoring in Germany. With further development, it should be possible to use a version of the GEDI with even stronger psychometric properties for area-wide monitoring of child development. Anonymous monitoring using an adapted, contextually appropriate GEDI in the preschool setting would have substantial reach into the target population, provide support to teachers in identifying problem areas, and at the same time facilitate target-oriented decision-making in public health policy.

## Supplementary information

**Additional file 1.** Bland-Altman plots with 95% limits of agreement for corresponding GEDI and SDQ (A) and DESK (B) domains, stratified by age groups.

## Data Availability

The datasets used and/or analyzed during the current study are available from the corresponding author upon reasonable request.

## Abbreviations

EDI: Early Development Instrument
GEDI: German version of the Early Development Instrument
PHY: Physical Health and Well-Being
PHY_3: Gross & Fine Motor Skills
SOC: Social Competence
SOC_1: Overall Social Competence With Peers
EMO: Emotional Maturity
EMO_1: Prosocial and Helping Behavior
EMO_3: Aggressive Behavior
EMO_4: Hyperactive and Inattentive Behavior
LAN: Language and Cognitive Development
LAN_1: Basic Literacy
COM: Communication and General Knowledge
DESK 3–6: Dortmund developmental screening for preschool 3 to 6 years
FMO: Fine Motors Skills
GMO: Gross Motor Skills
SZK: Social Competence
SZV: Social Behavior
SZI: Social Interaction
AKN: Attention and Concentration
KSP: Cognition and Language
KOG: Cognition
BKS: Basic Competence Literacy
BKM: Basic Competence Numeracy
SPK: Language and Communication
SDQ: Strengths and Difficulties Questionnaire
SDQ_peers: Peer Problems Scale
SDQ_prosocial: Prosocial Scale
SDQ_conduct: Conduct Problems Scale
SDQ_hyperactivity: Hyperactivity Scale
EFA: Exploratory Factor Analysis
IRT: Item Response Theory
OR: Odds Ratio
SES: Socioeconomic Status
SOPESS: Social Pediatric Developmental Screening for School Entry Examinations
