# Supplementary material for: Psychometric properties and contextual appropriateness of the German version of the Early Development Instrument
Source: BMC Pediatr. 2020 Jul 9;20:339. doi: 10.1186/s12887-020-02191-w (PMC7346437; doi:10.1186/s12887-020-02191-w)
Supplement: Supplementary file 1 — Additional file 1. Bland-Altman plots with 95% limits of agreement for corresponding GEDI and SDQ (A) and DESK (B) domains, stratified by age groups. [file 12887_2020_2191_MOESM1_ESM.docx]

**Bland Altman plots**

**A – Bland Altman Plots with 95% limits of agreement for corresponding GEDI and SDQ domains, stratified by age groups**

| **Figure 1: GEDI domain SOC_1 (overall social competence with peers) and SDQ domain peers (peer relationship problems)** | |
| --- | --- |
| 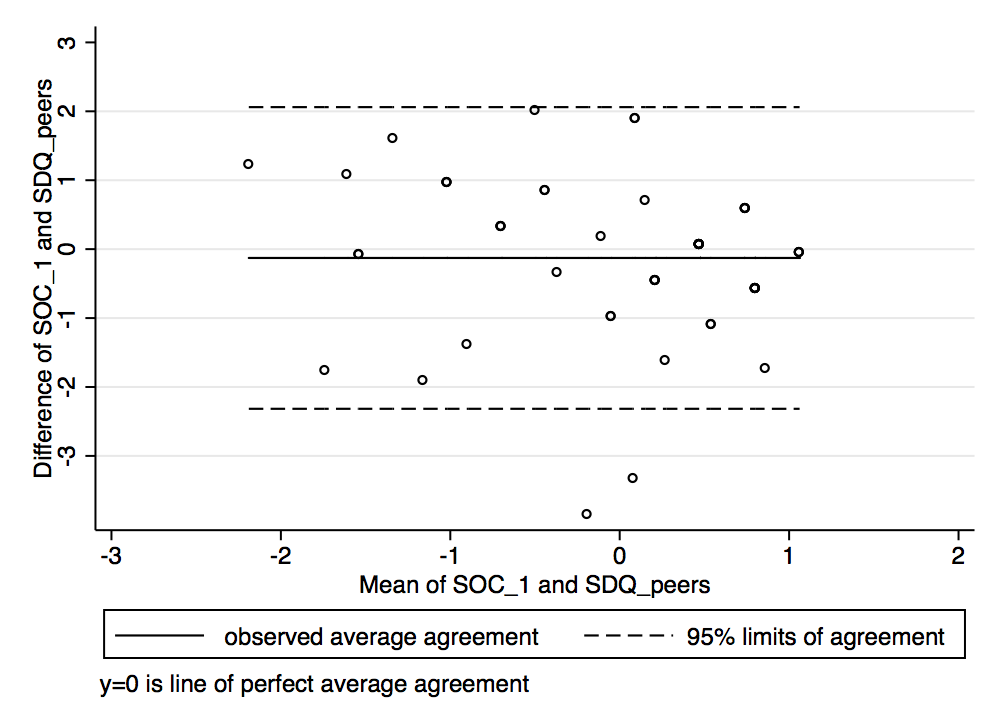  A) age 3 [95% limits of agreement: -2.32 to 2.06] | **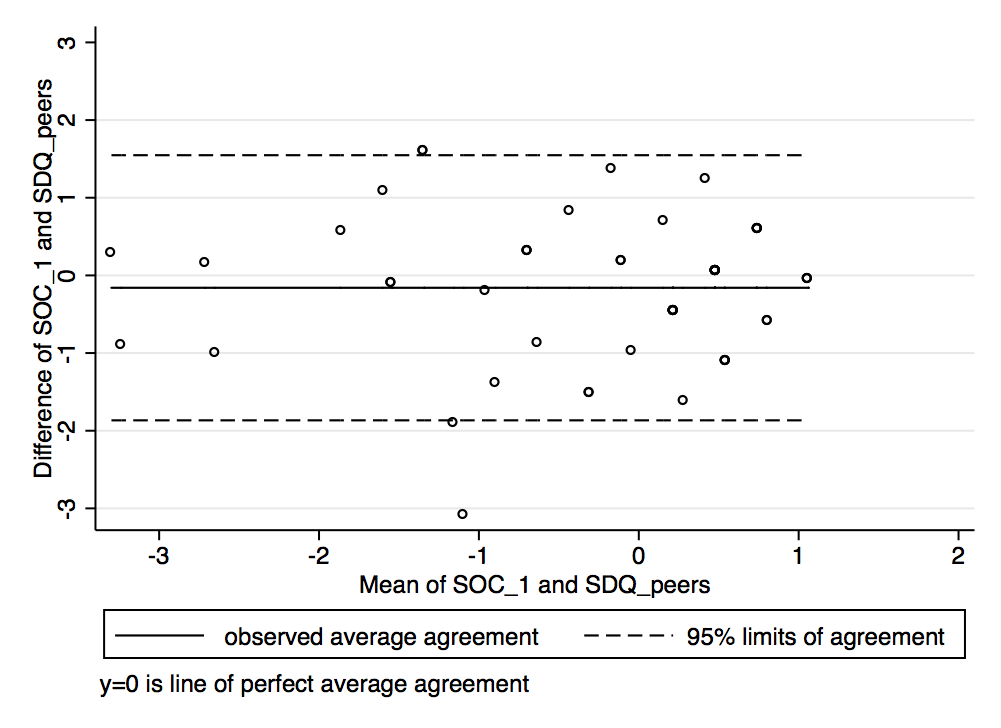**  B) age 4 [95% limits of agreement: -1.87 to 1.55] |
| 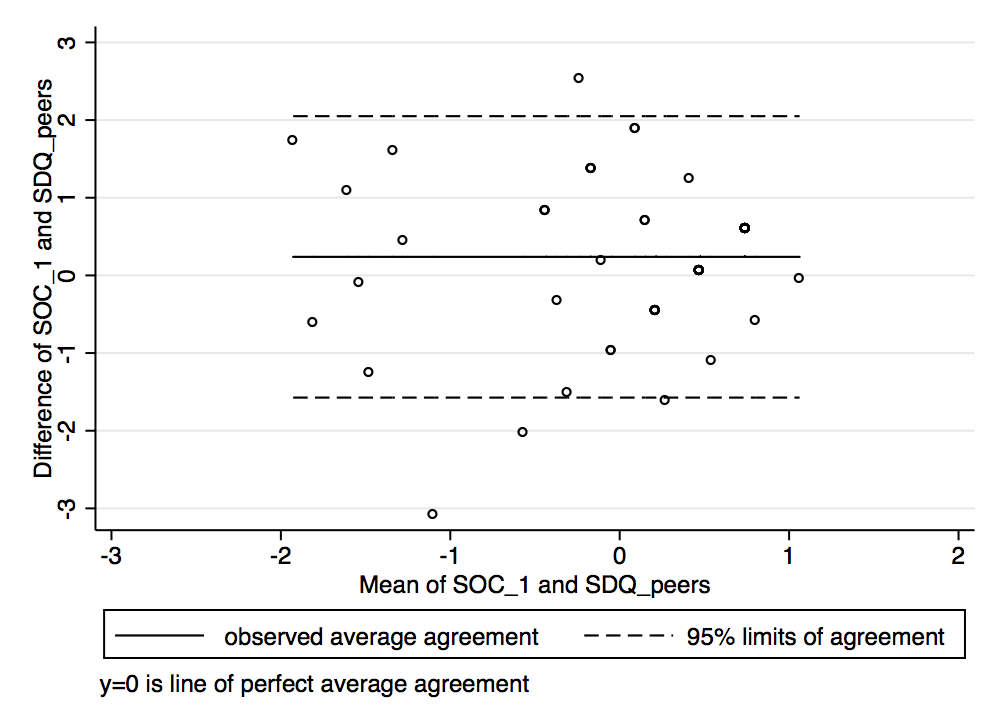  C) ages 5 & 6 [95% limits of agreement: -1.57 to 2.05] | 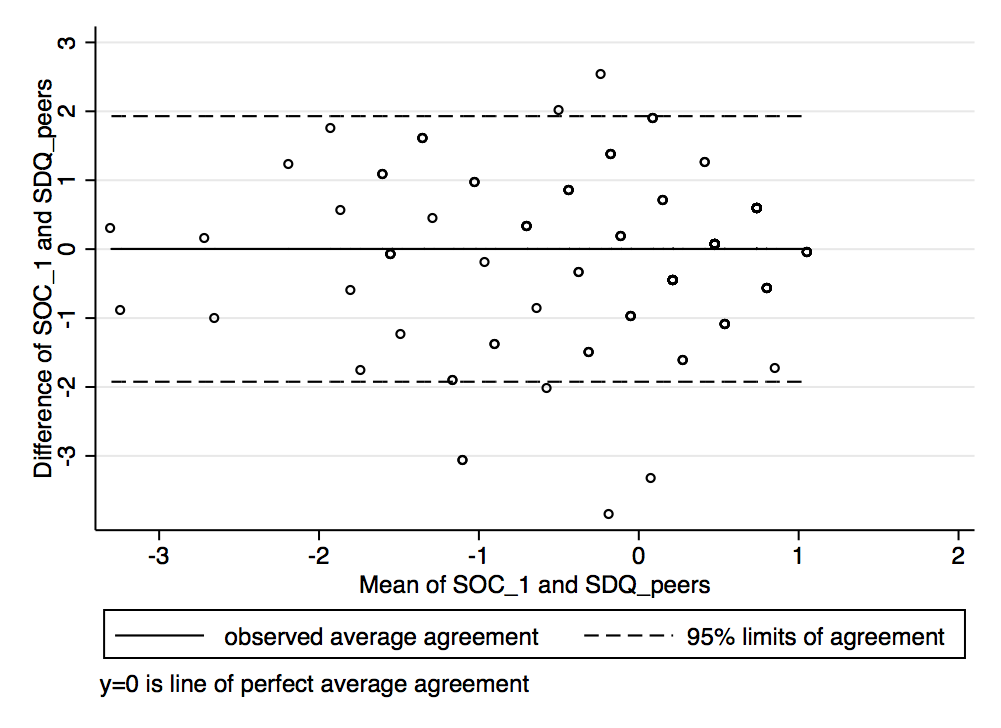  D) overall sample [95% limits of agreement -1.92 to 1.93] |

| **Figure 2: GEDI domain EMO_1 (prosocial and helping behavior) and SDQ domain peers (peer relationship problems)** | |
| --- | --- |
| 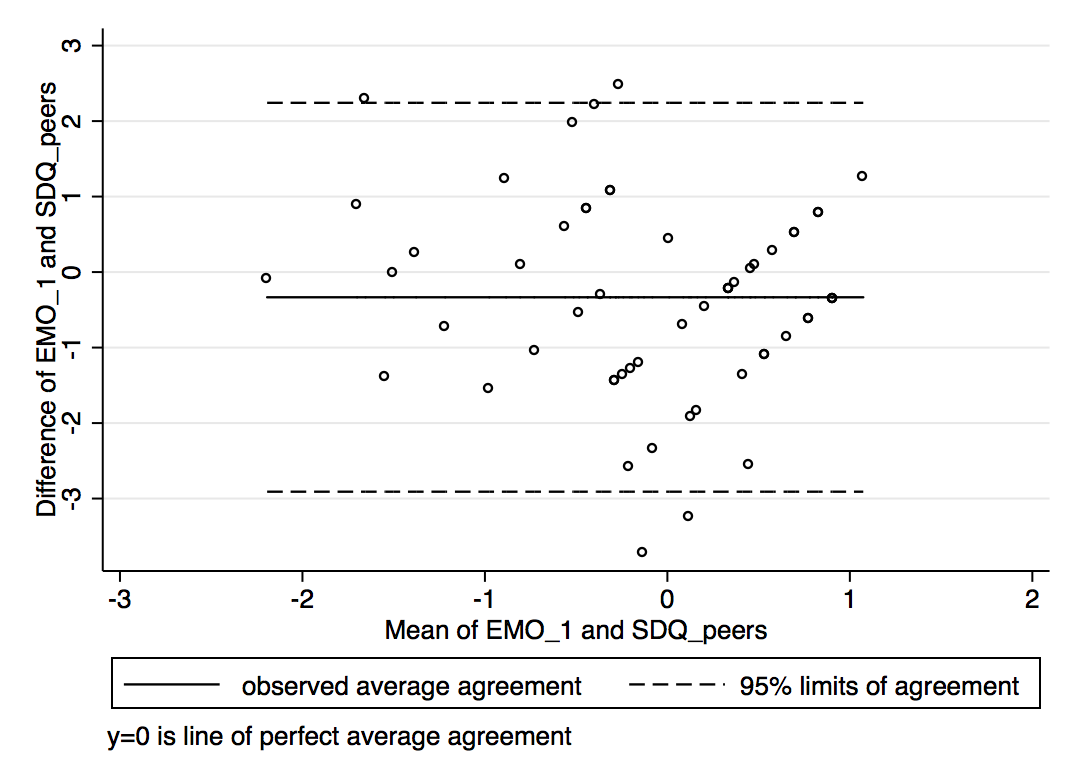  A) age 3 [95% limits of agreement: -2.91 to 2.24] | **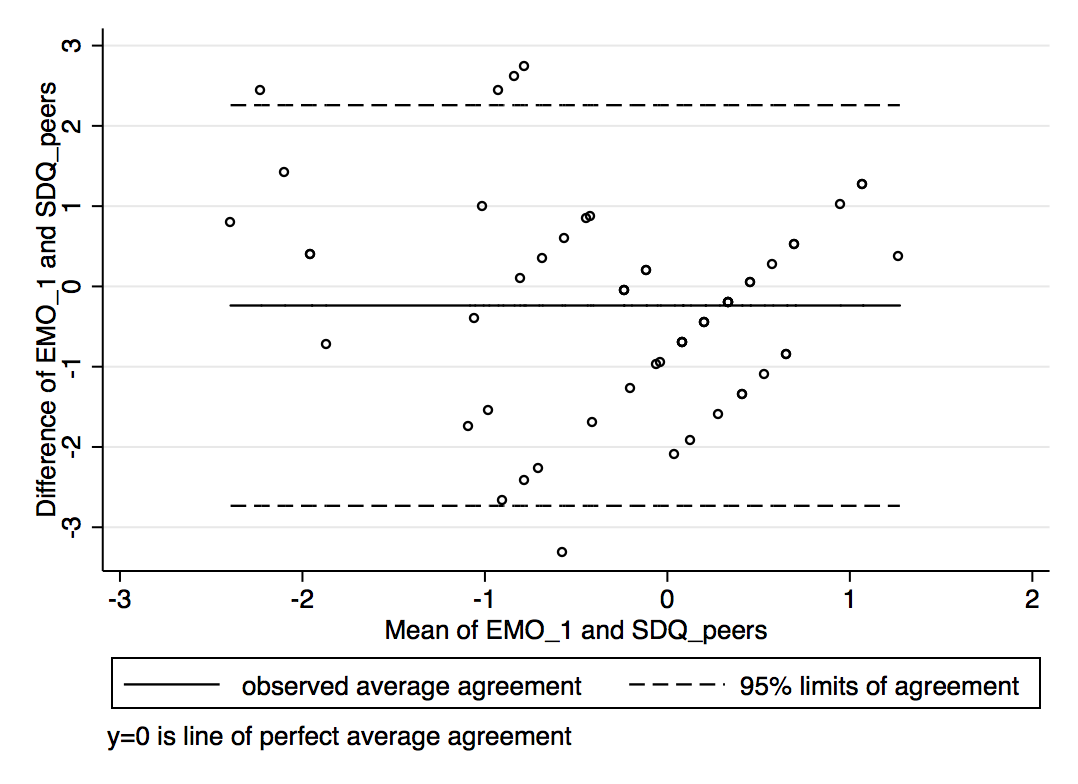**  B) age 4 [95% limits of agreement: -2.73 to 2.26] |
| 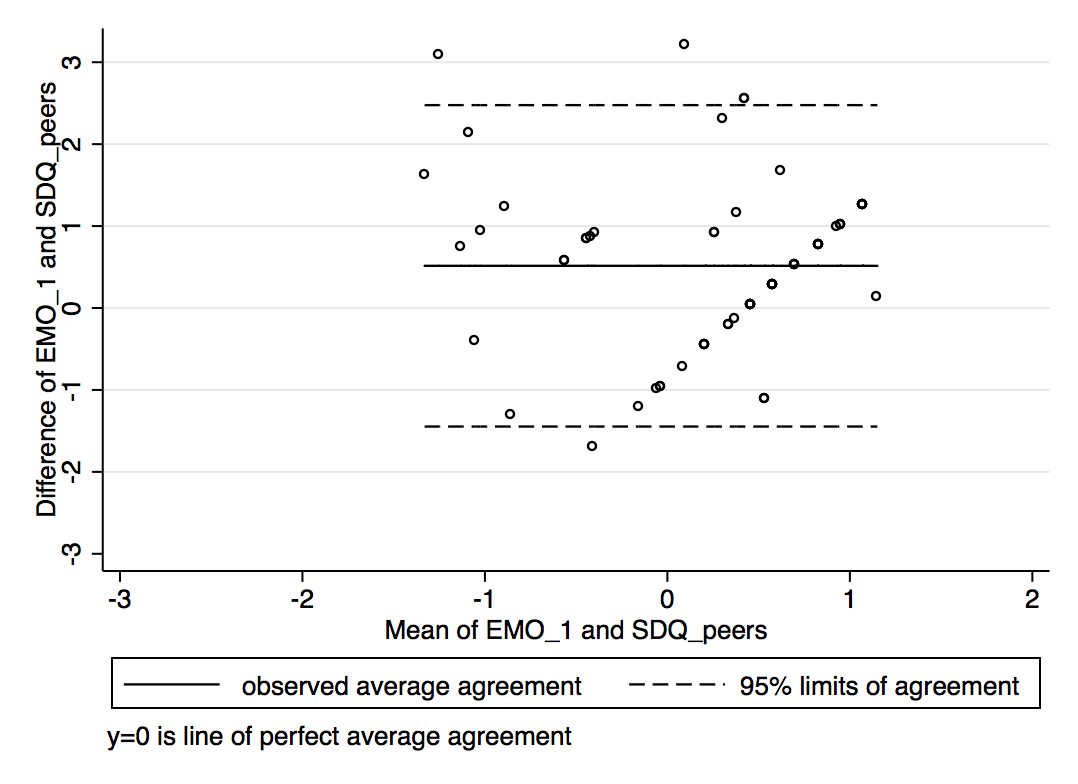  C) ages 5 & 6 [95% limits of agreement: -1.45 to 2.48] | 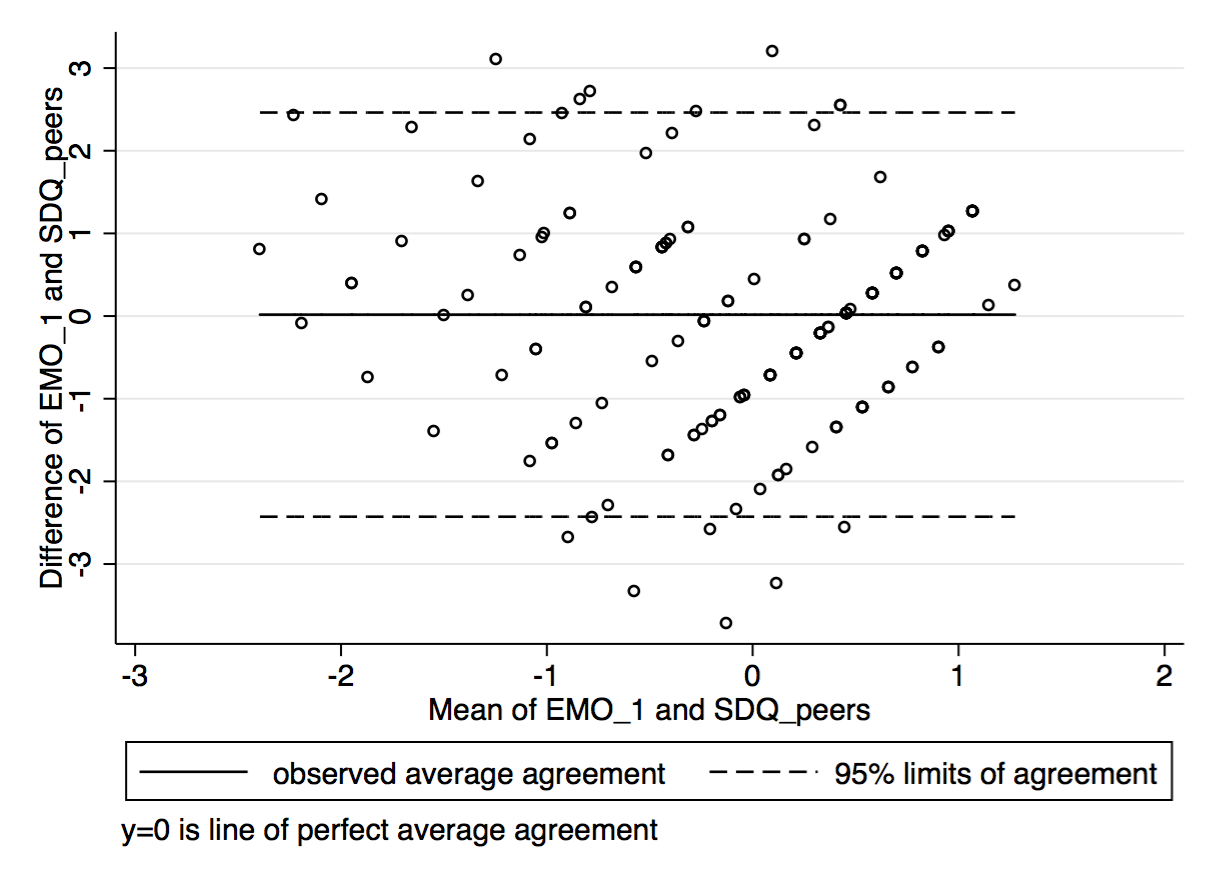  D) overall sample [95% limits of agreement -2.43 to 2.46] |

| **Figure 3: GEDI domain EMO_1 (psosocial and helping behavior) and SDQ domain prosoc (prosocial behaviour)** | |
| --- | --- |
| 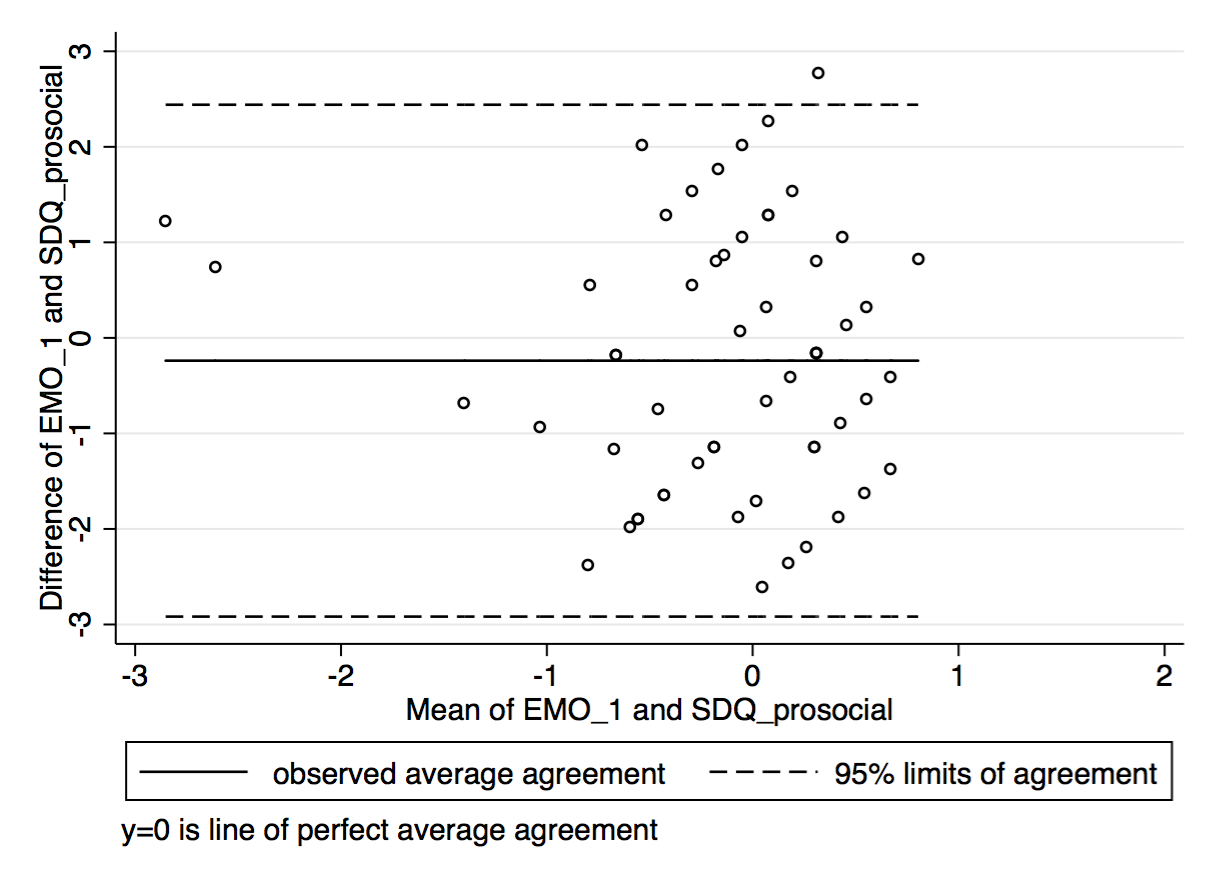  A) age 3 [95% limits of agreement: -2.92 to 2.44] | **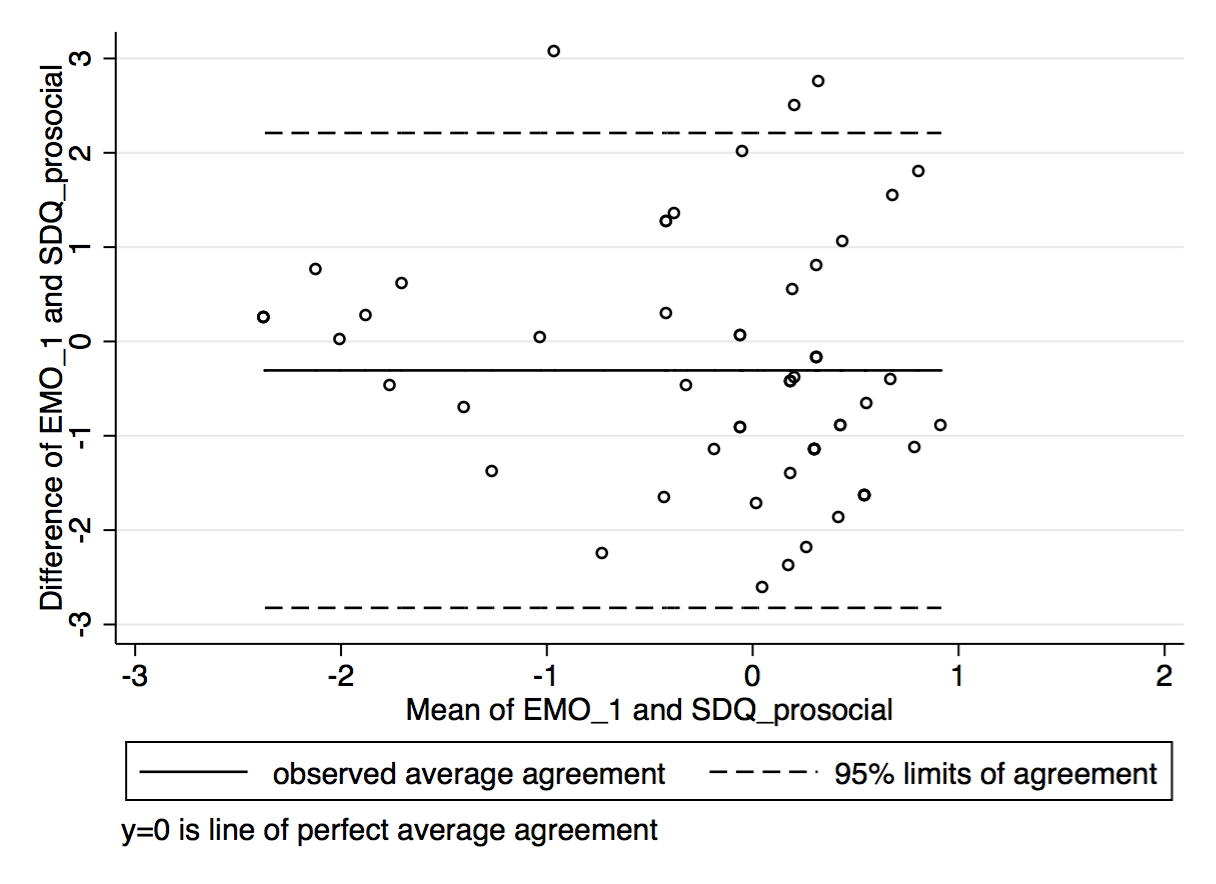**  B) age 4 [95% limits of agreement: -2.82 to 2.21] |
| 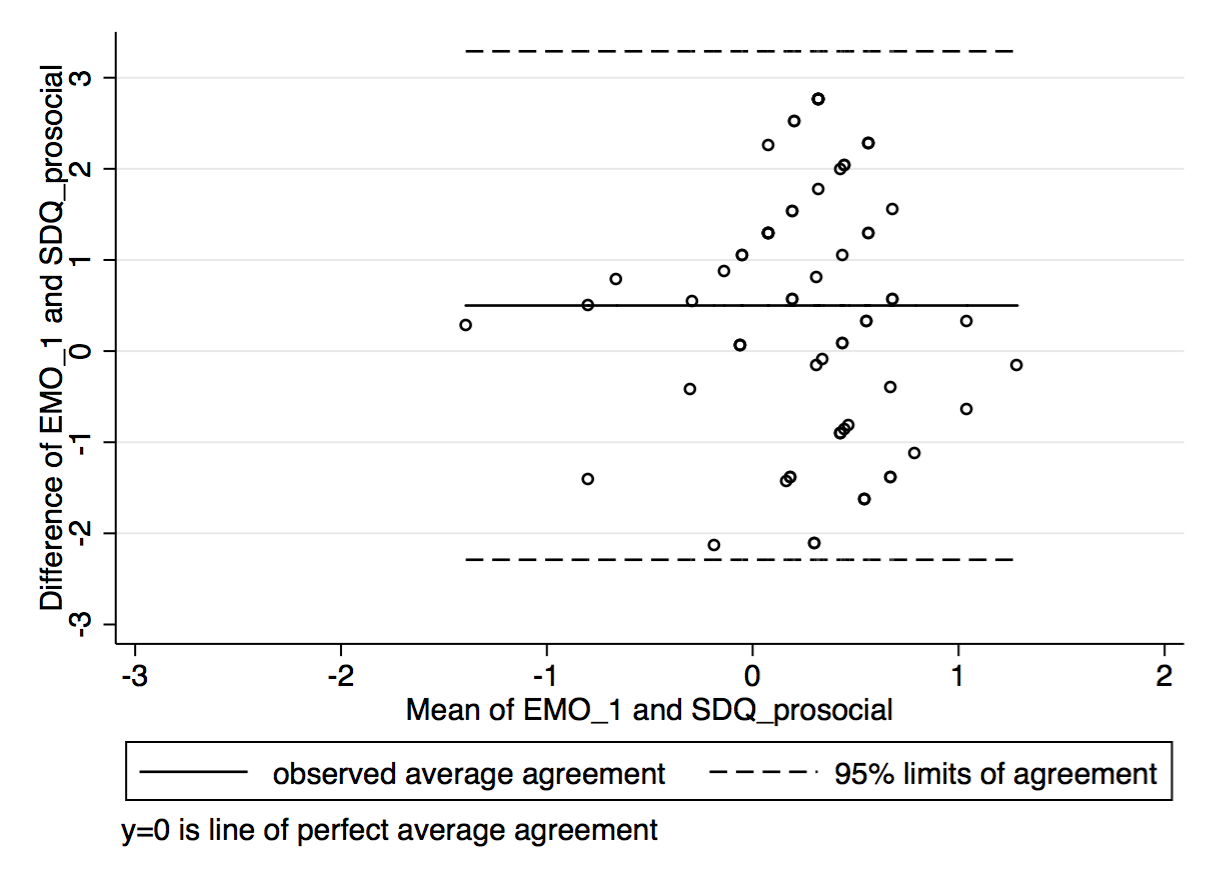  C) ages 5 & 6 [95% limits of agreement: -2.29 to 3.29] | 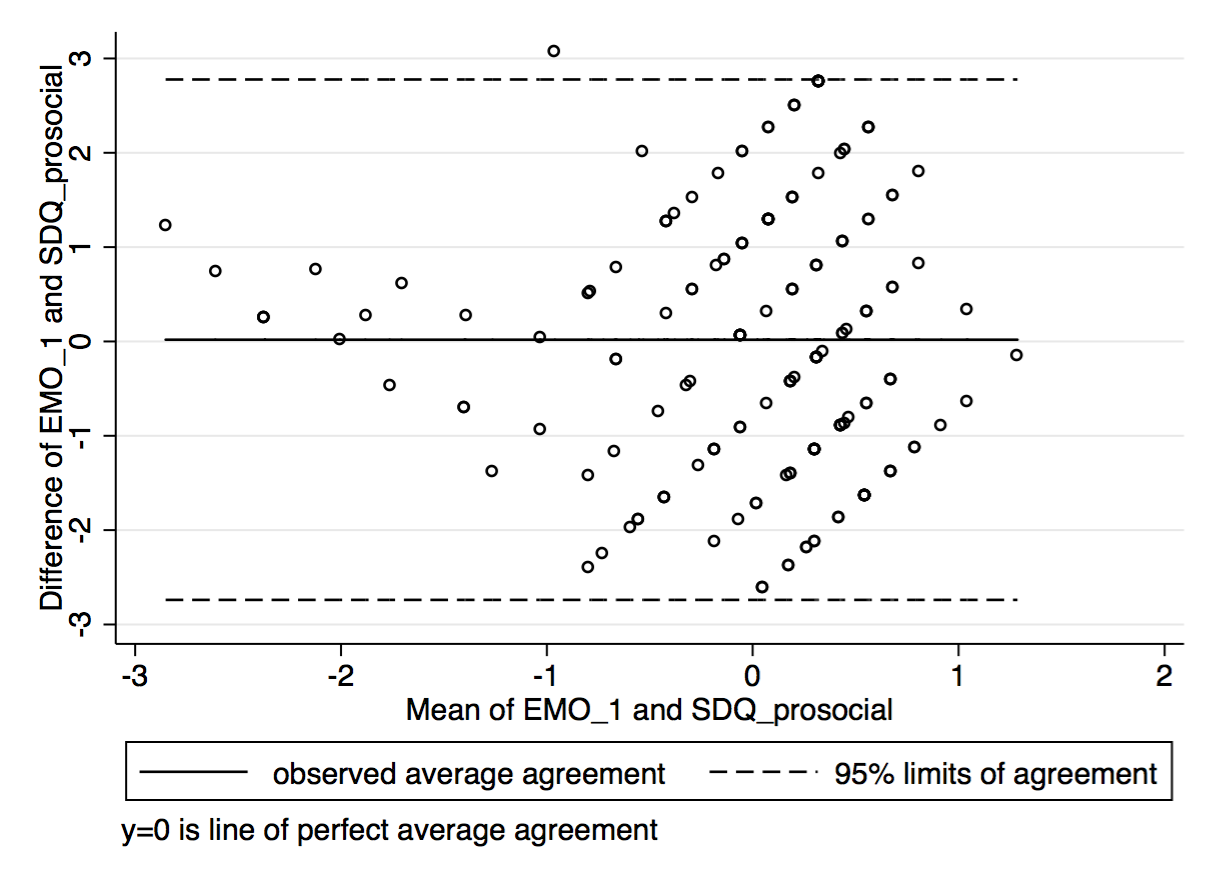  D) overall sample [95% limits of agreement -2.74 to 2.78] |

| **Figure 4: GEDI domain EMO_3 (aggressive behaviour) and SDQ domain conduct (conduct problems)** | |
| --- | --- |
| 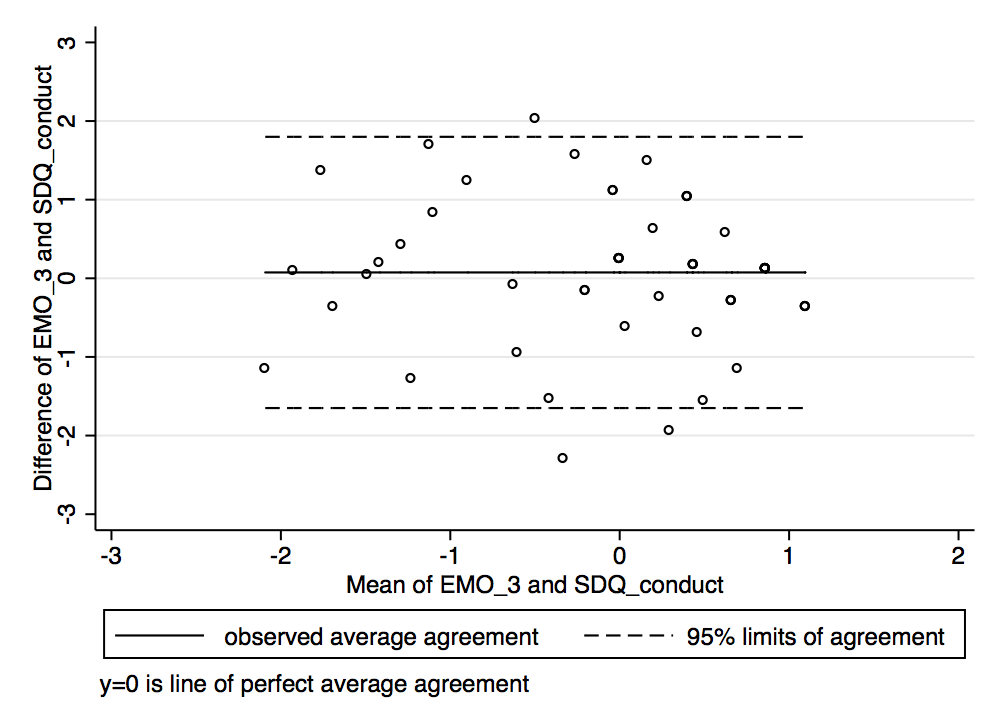  A) age 3 [95% limits of agreement: -1.65 to 1.8] | **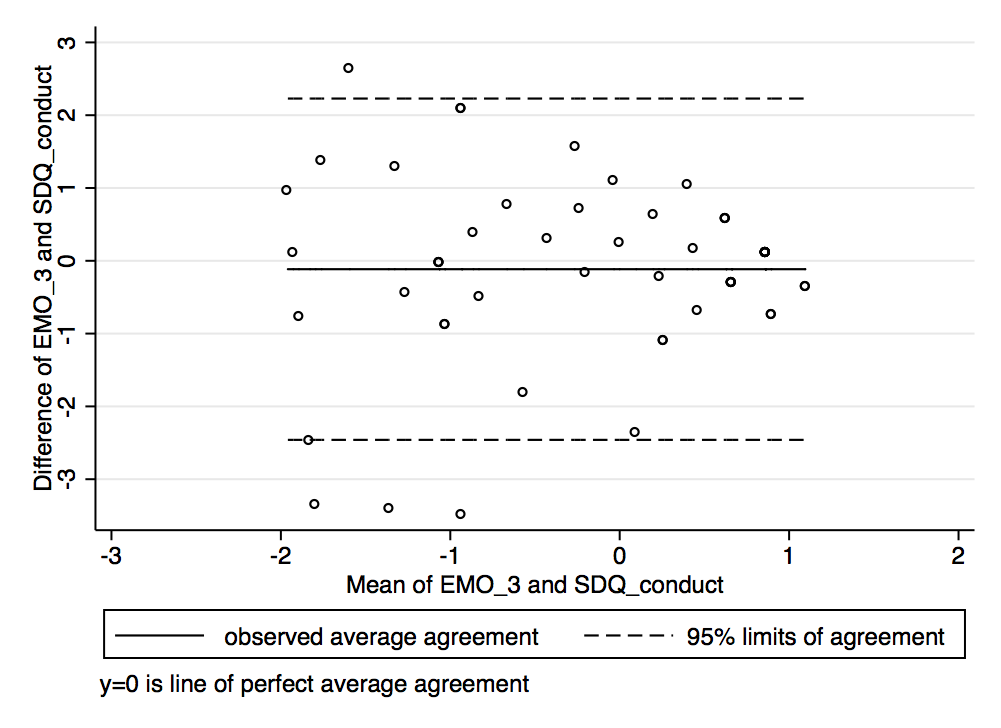**  B) age 4 [95% limits of agreement: -2.46 to 2.23] |
| 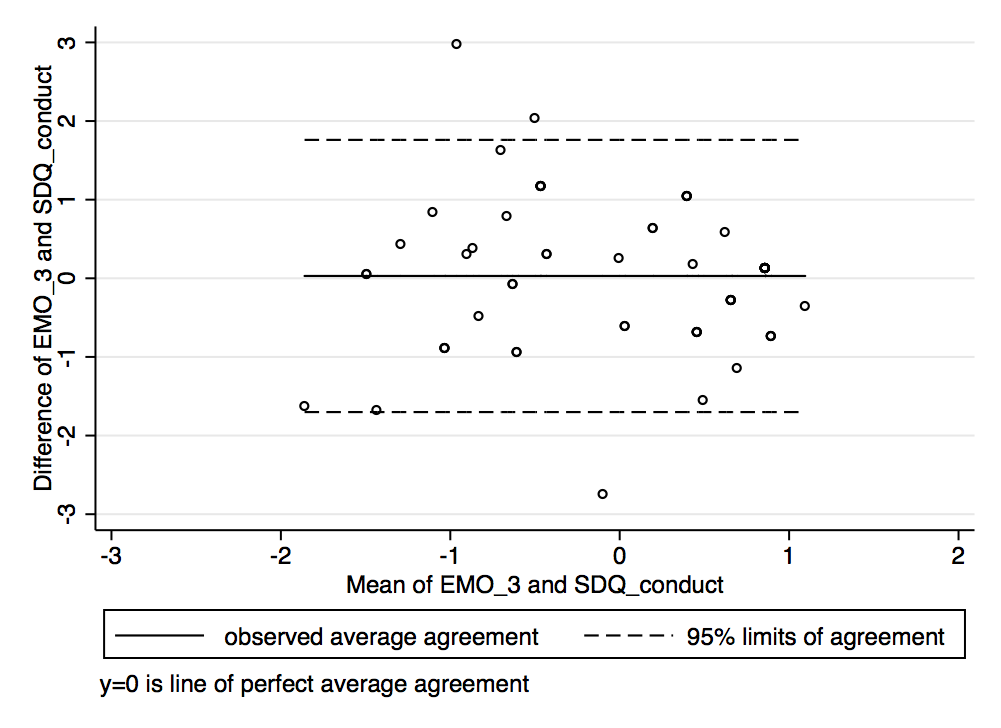  C) ages 5 & 6 [95% limits of agreement: -1.70 to 1.76] | 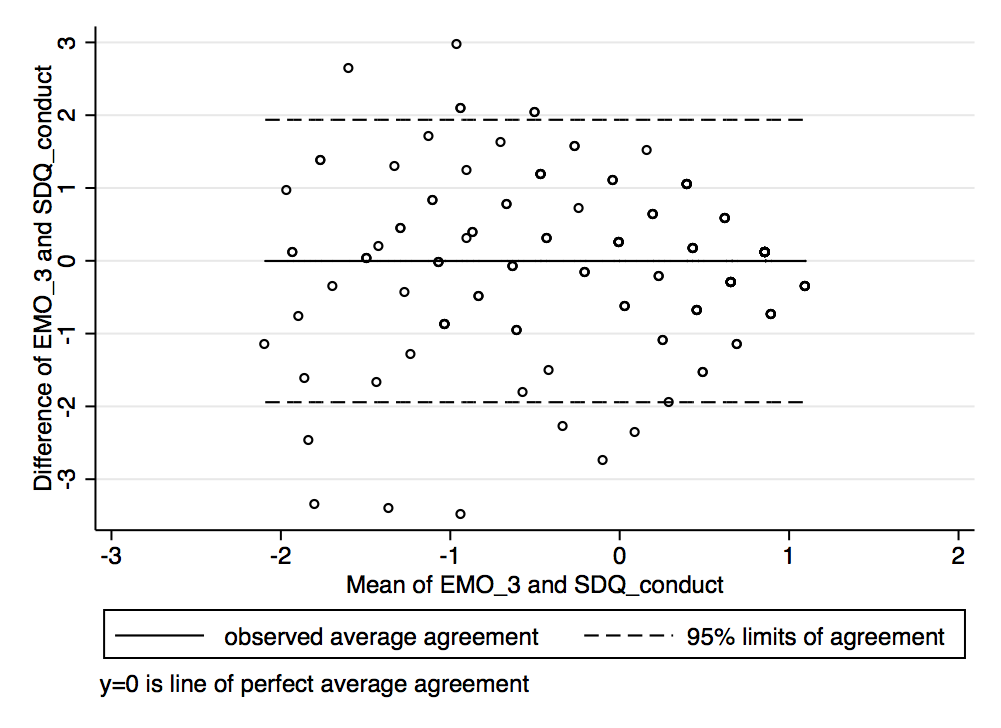  D) overall sample [95% limits of agreement -1.94 to 1.94] |

| **Figure 5: GEDI domain EMO_4 (hyperactive and inattentive behaviour) and SDQ domain hyper (hyperactivity/inattention)** | |
| --- | --- |
| 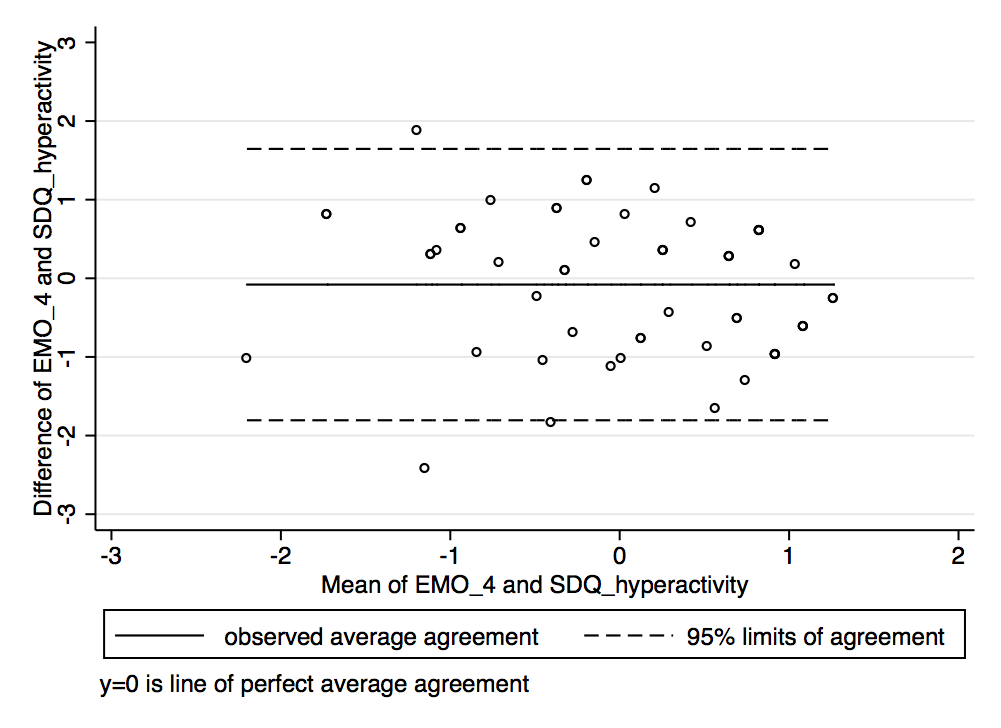  A) age 3 [95% limits of agreement: -1.81 to 1.65] | **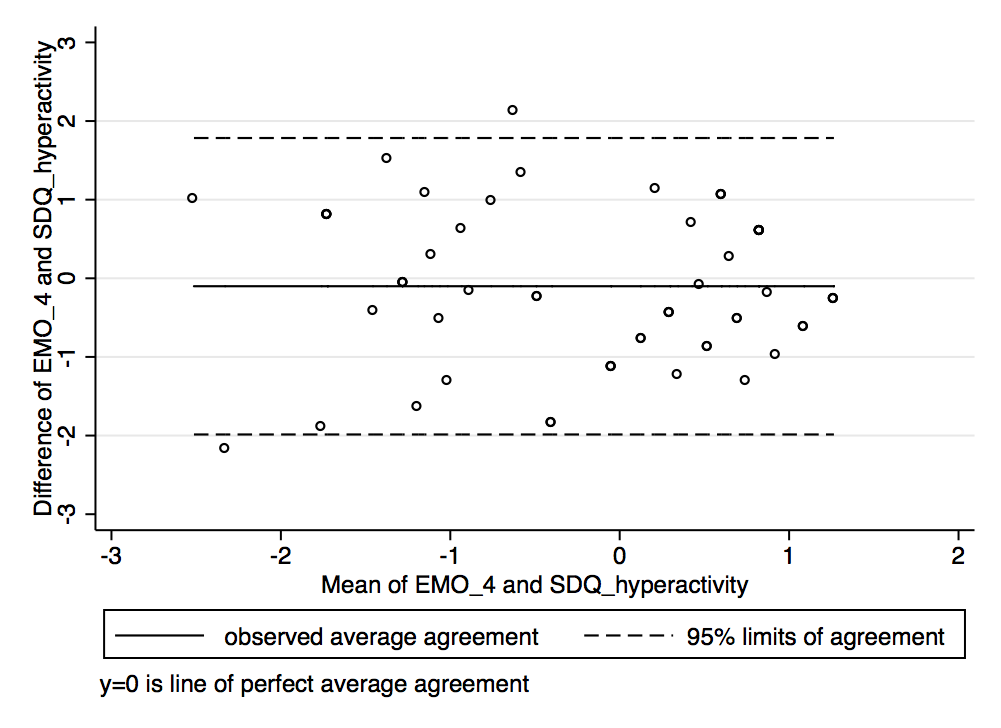**  B) age 4 [95% limits of agreement: -1.99 to 1.78] |
| 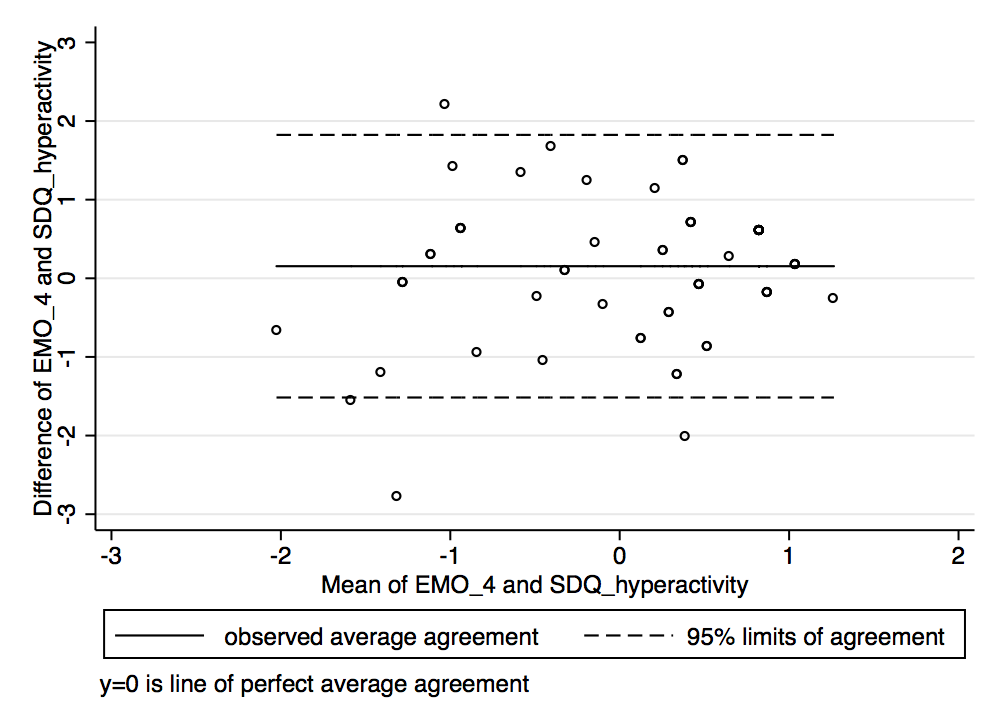  C) ages 5 & 6 [95% limits of agreement: -1.52 to 1.82] | 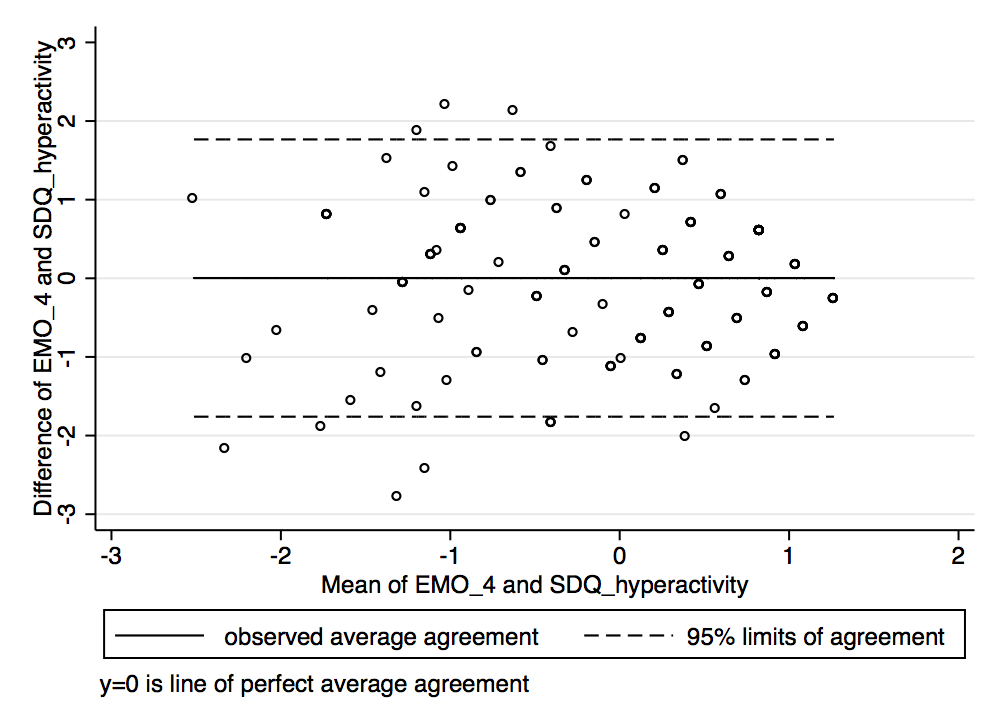  D) overall sample [95% limits of agreement -1.76 to 1.77] |

**B – Bland Altman Plots with 95% limits of agreement for corresponding GEDI and DESK domains, stratified by age groups**

| **Figure 1: GEDI domain PHY_3 (gross & fine motor skills) and DESK domain FMO (fine motor skills)** | |
| --- | --- |
| 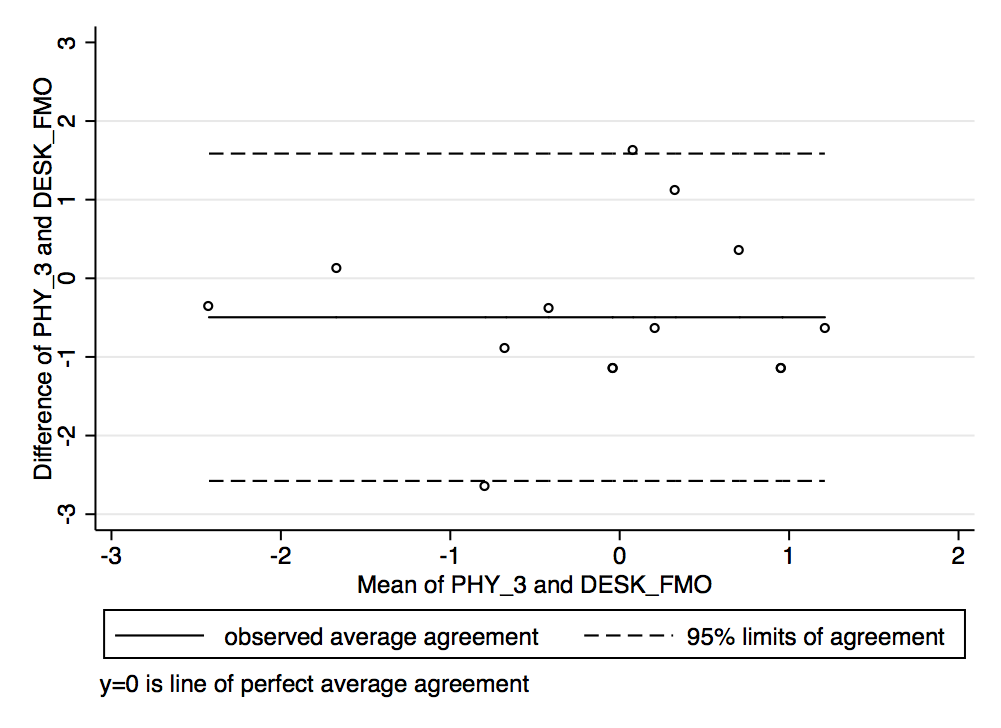  A) age 3 [95% limits of agreement: -2.58 to 1.59] | **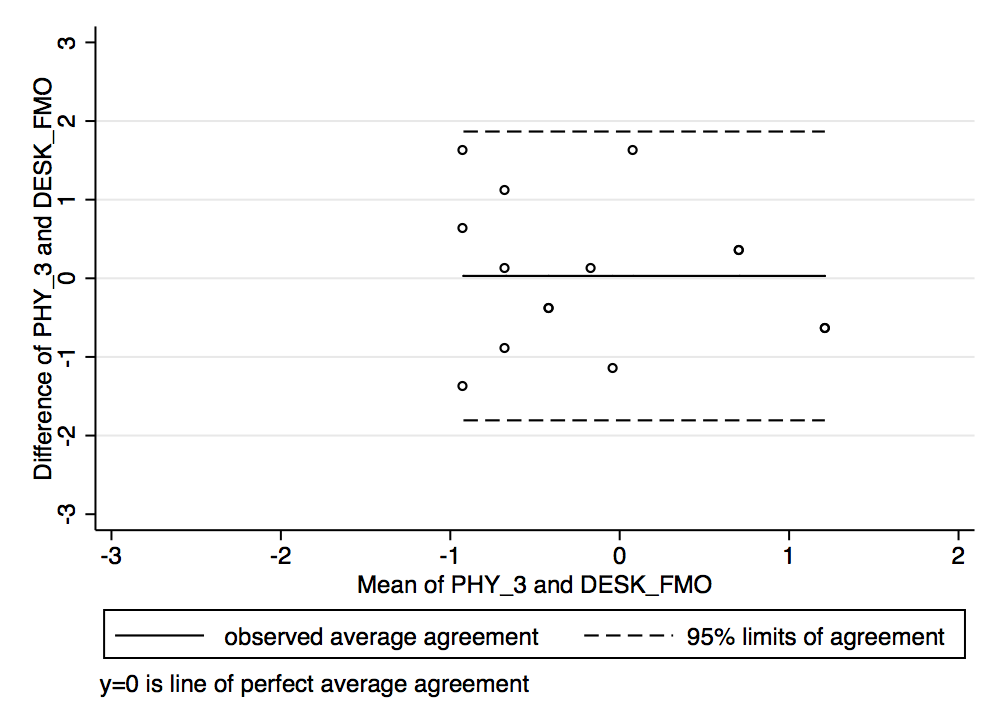**  B) age 4 [95% limits of agreement: -1.81 to 1.87] |
| 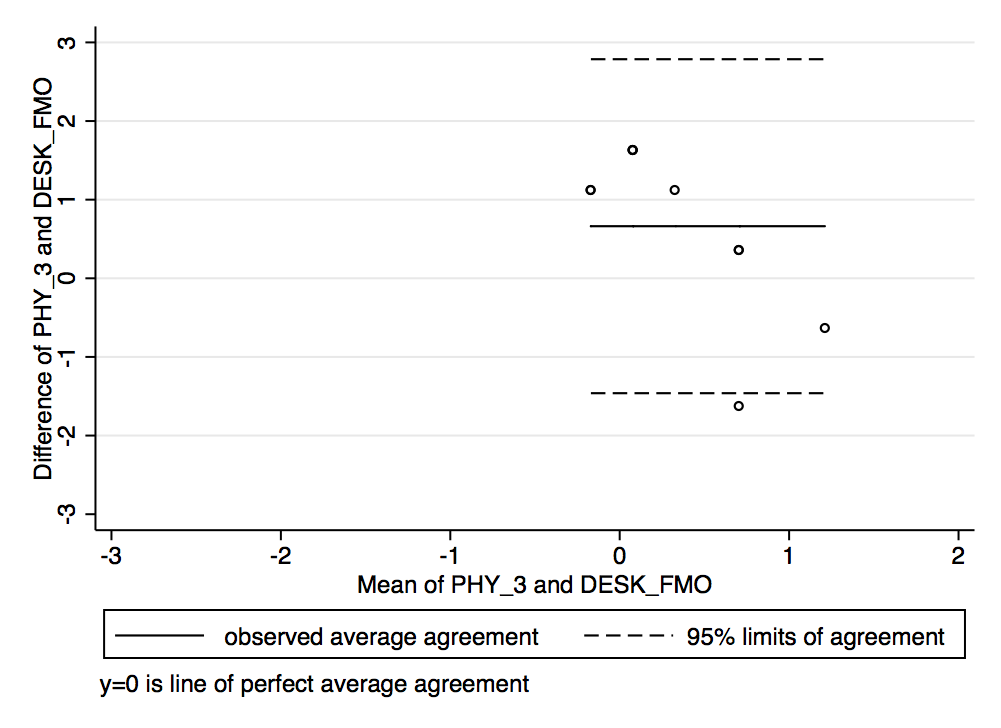  C) ages 5 & 6 [95% limits of agreement: -1.46 to 2.79] | 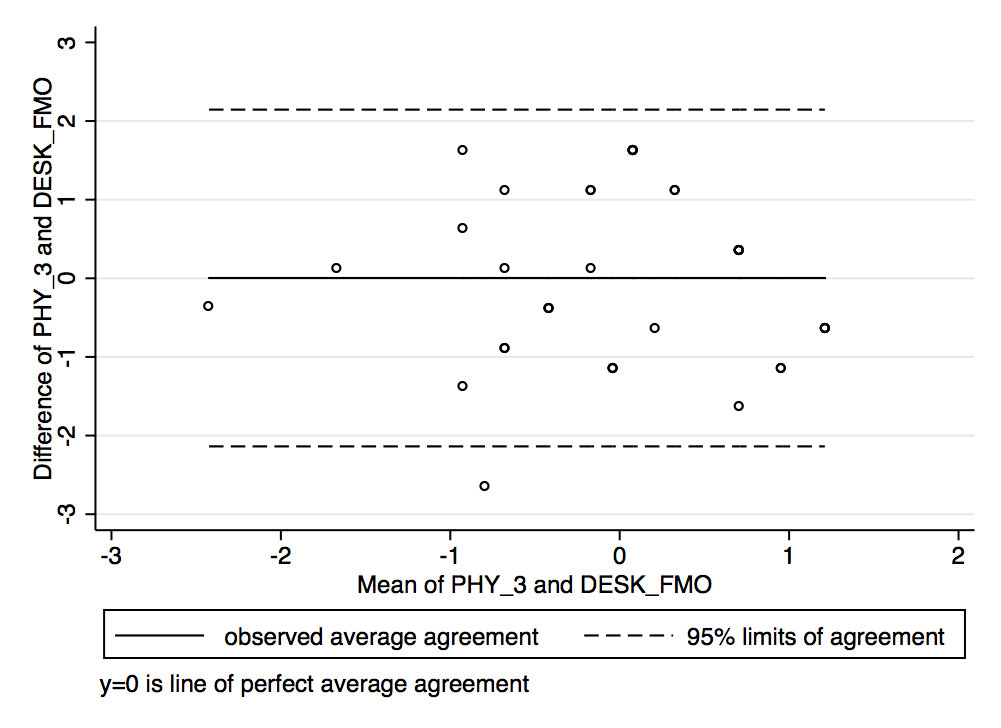  D) overall sample [95% limits of agreement -2.14 to 2.15] |

| **Figure 2: GEDI domain PHY_3 (gross & fine motor skills) and DESK domain GMO (gross motor skills)** | |
| --- | --- |
| 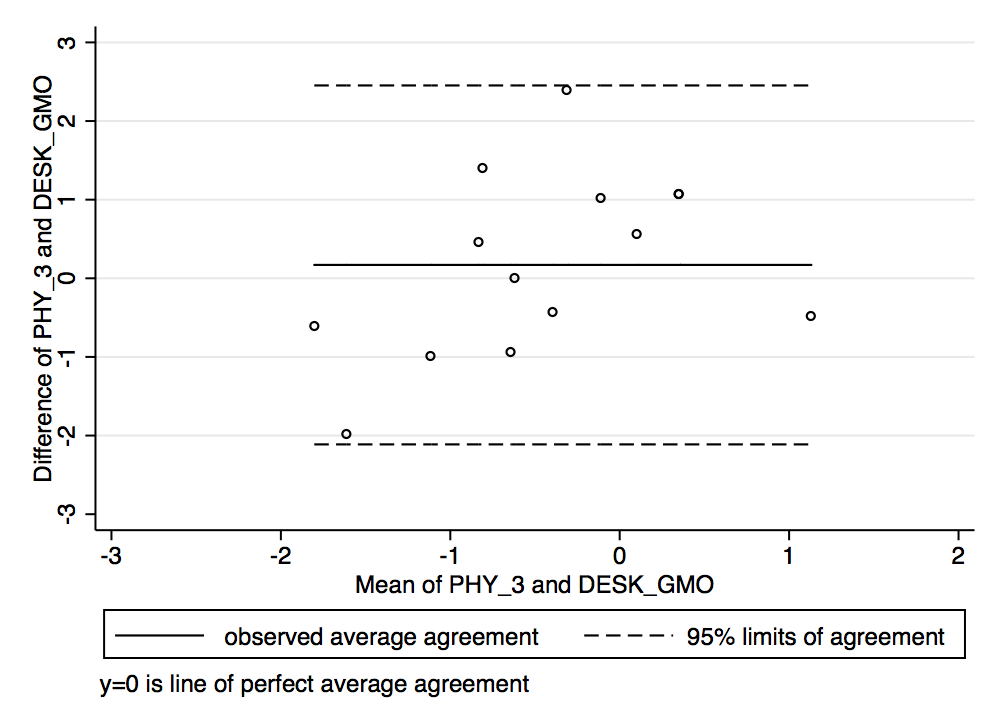  A) age 3 [95% limits of agreement: -2.11 to 2.45] | **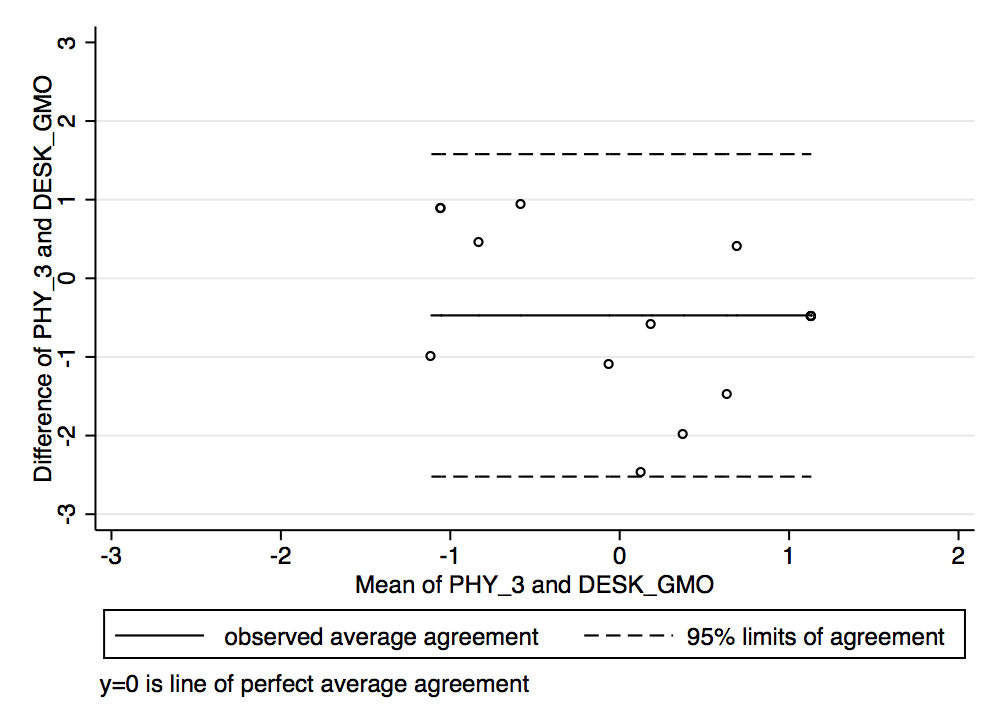**  B) age 4 [95% limits of agreement: -2.52 to 1.58] |
| 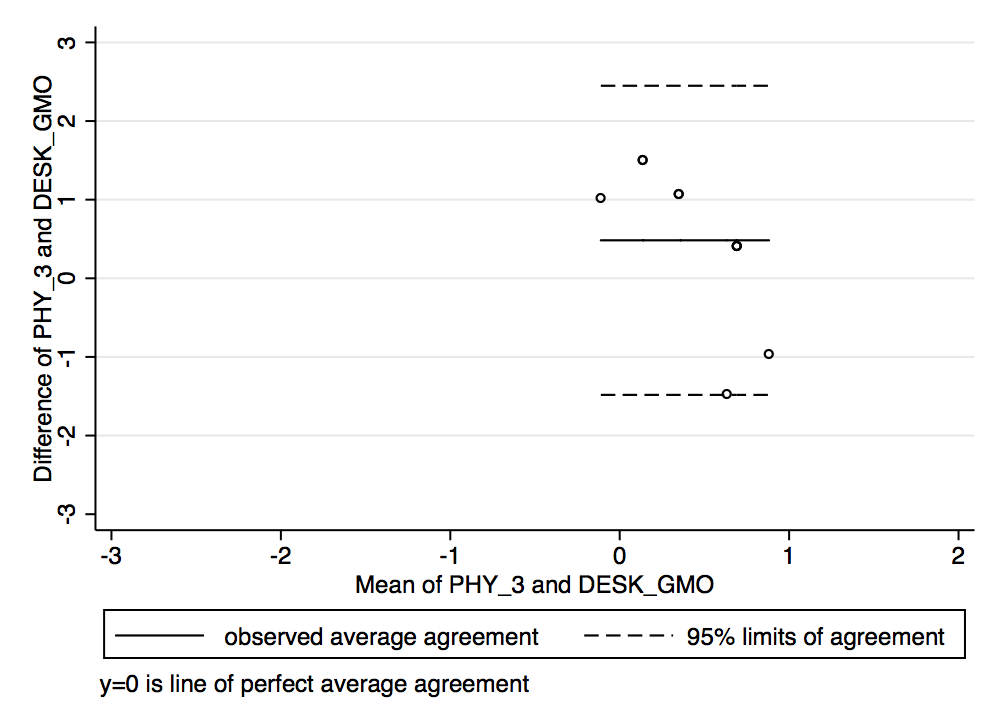  C) ages 5 & 6 [95% limits of agreement: 1.48 to 2.45] | 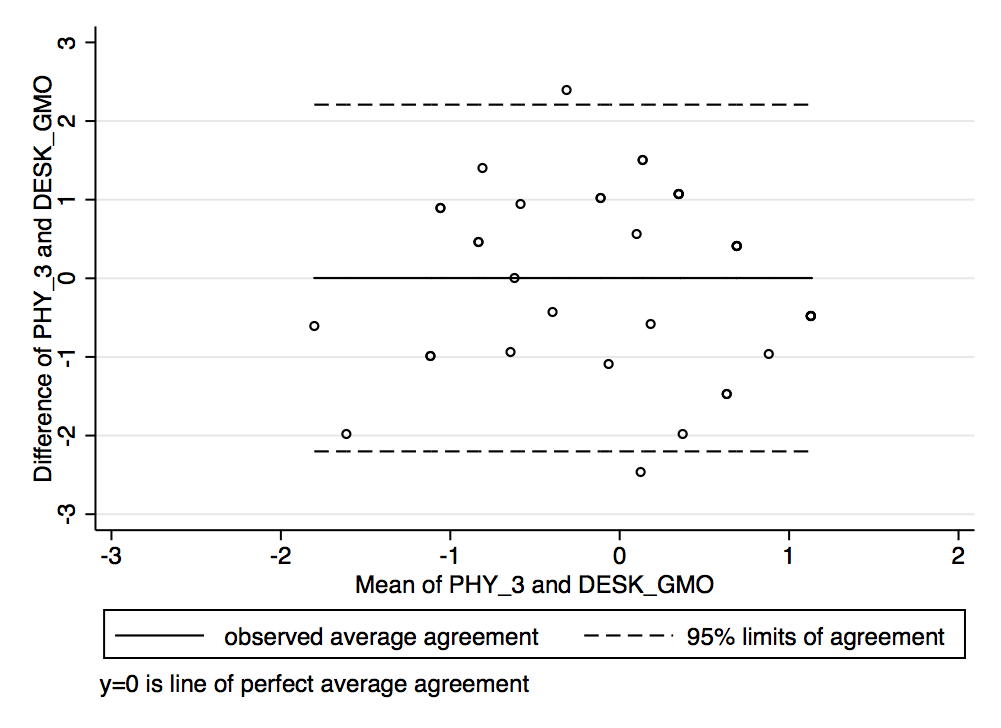  D) overall sample [95% limits of agreement -2.20 to 2.21] |

| **Figure 3: GEDI domain SOC_1 (overall social competence with peers) and DESK domain SZV (social behaviour)** | |
| --- | --- |
| 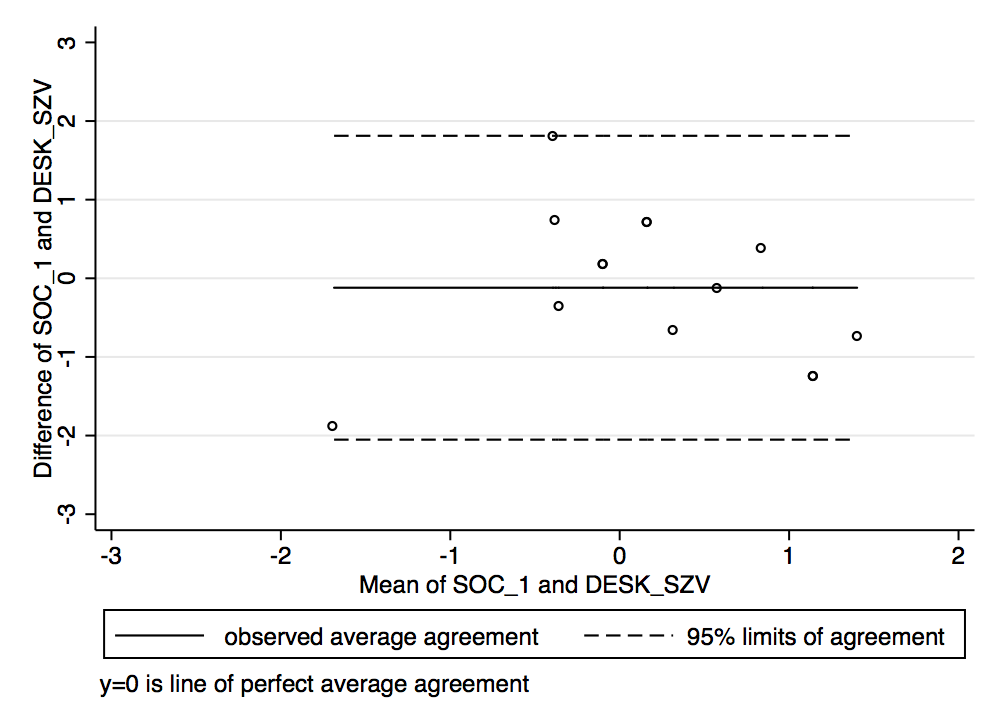  A) age 3 [95% limits of agreement: -2.05 to 1.81] | 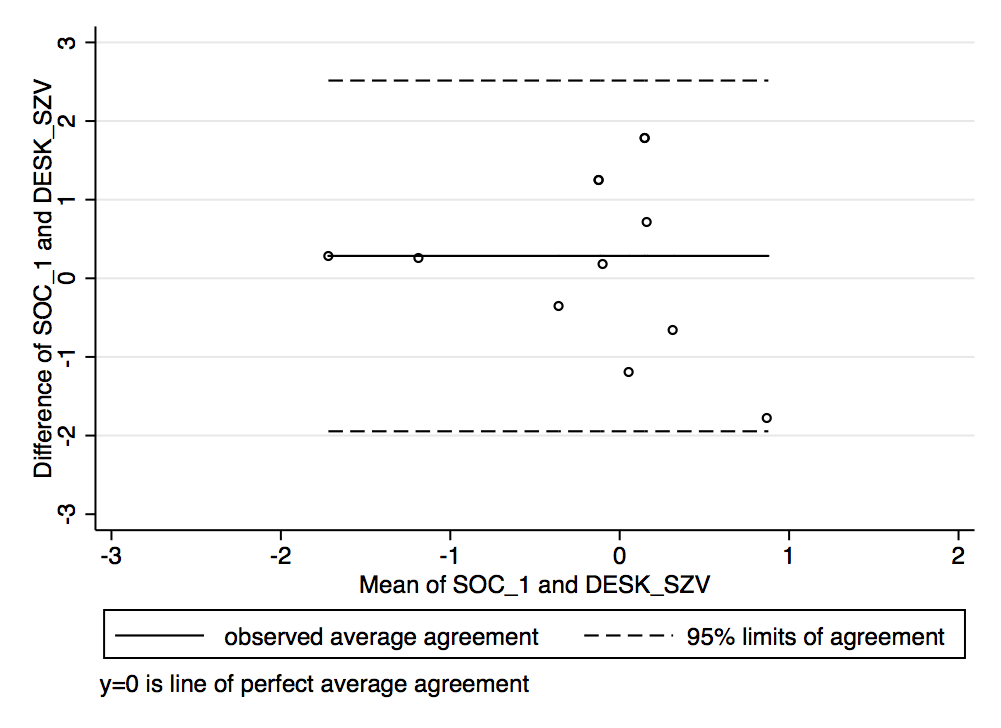  B) age 4 [95% limits of agreement: -1.95 to 2.52] |
| C) ages 5 & 6  *Questionnaires for 5 and 6 year old children do not include the domain “social behavior”* | 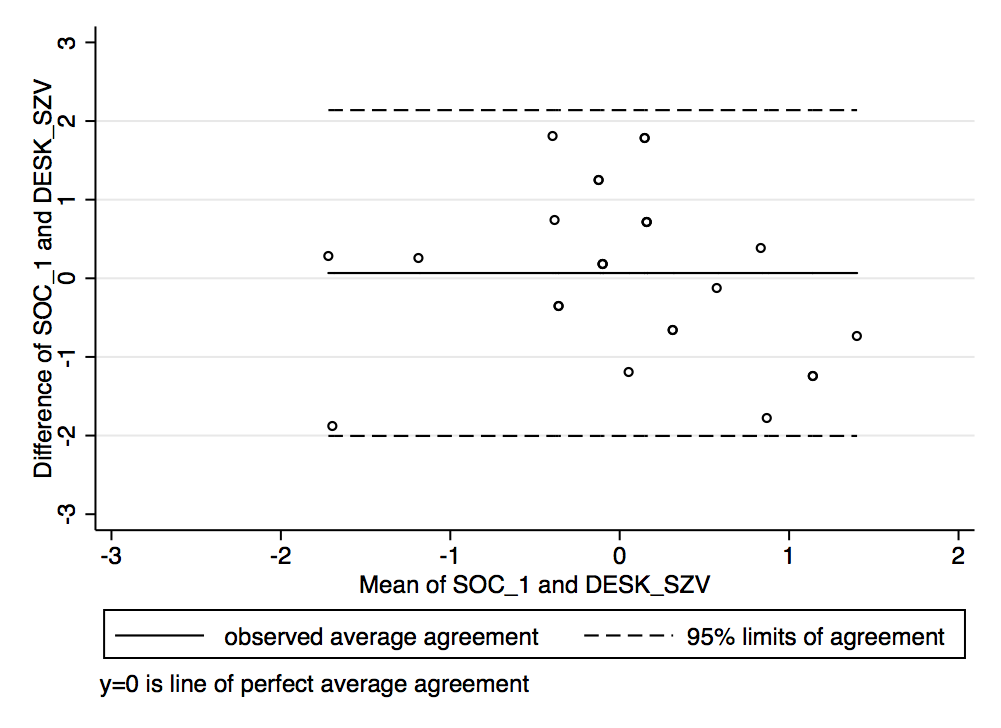  D) overall sample [95% limits of agreement -2.01 to 2.14] |

| **Figure 4: GEDI domain LAN_1 (basic literacy) and DESK domain KOG (cognition)** | |
| --- | --- |
| A) *Questionnaire for 3 year old children does not include the domain “cognition”* | 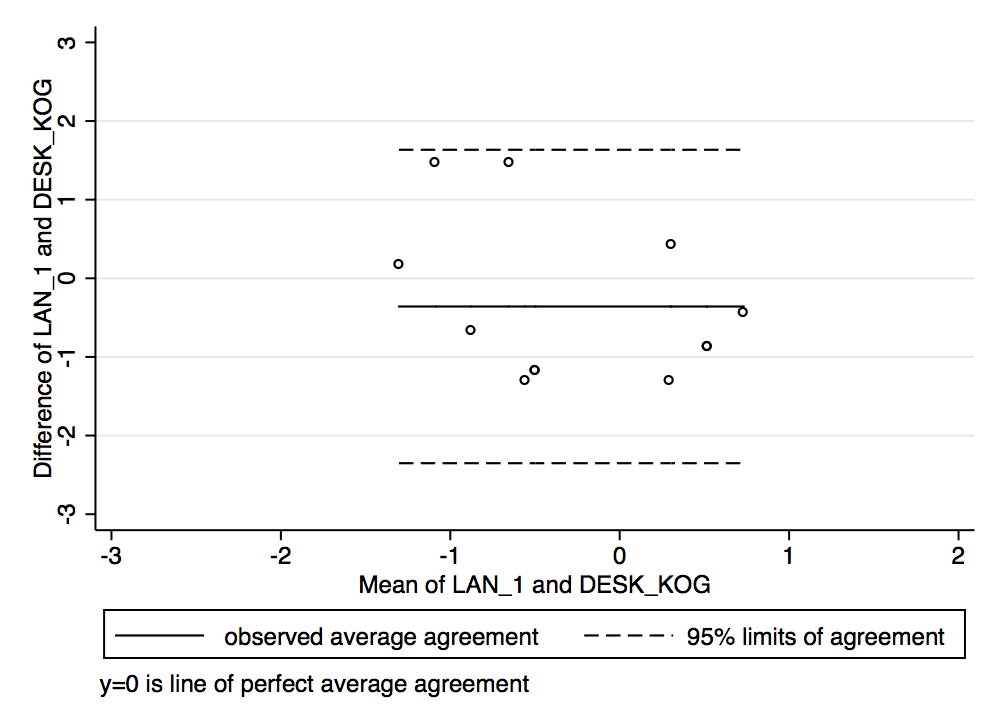  B) age 4 [95% limits of agreement: -2.35 to 1.63] |
| C) *Questionnaire for 5 and 6 year old children does not include the domain “cognition”* | 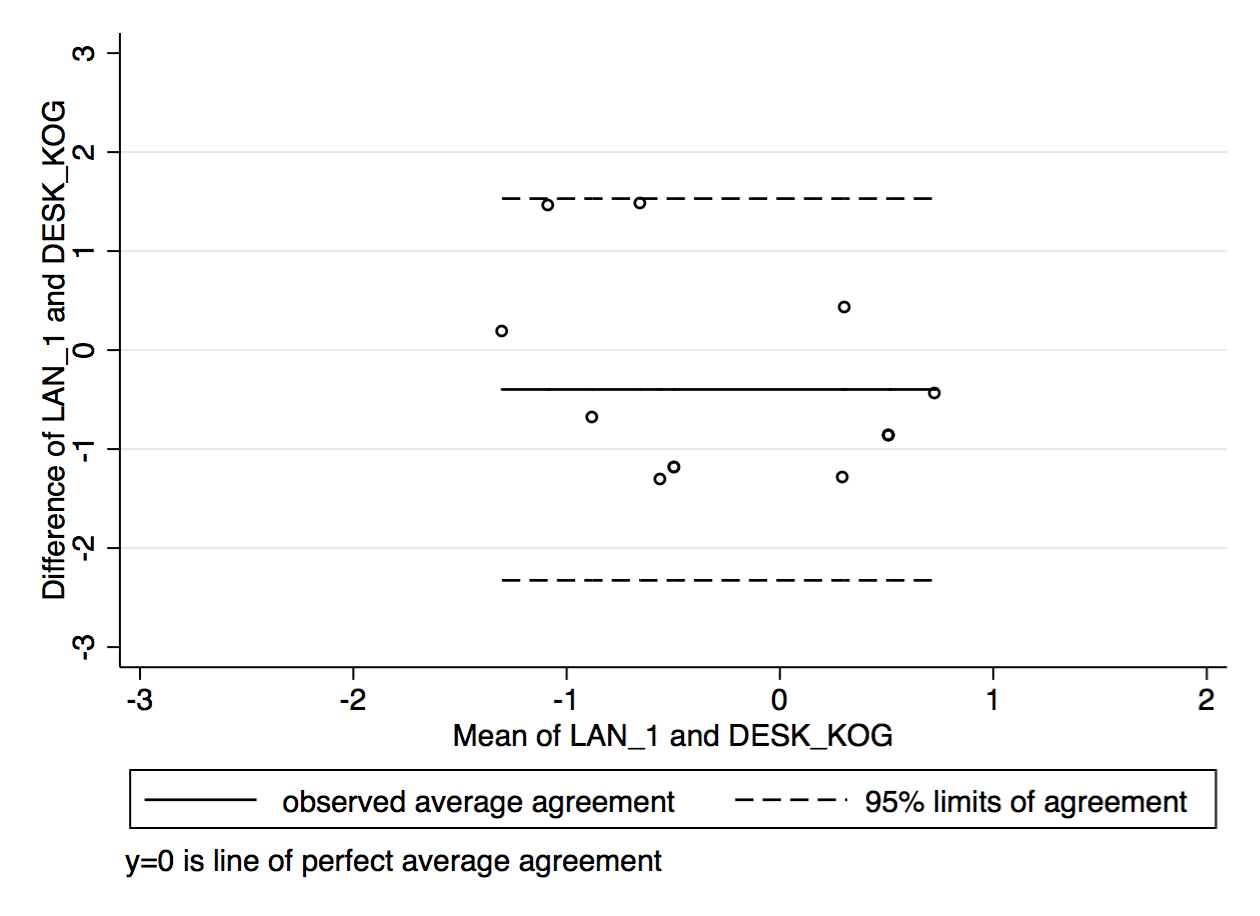  D) overall sample [95% limits of agreement -2.33 to 1.53] |

| **Figure 5: GEDI domain COM (communication and general knowledge) and DESK domain SPK (language and communication)** | |
| --- | --- |
| A) *Questionnaire for 3 year old children does not include the domain “language and communication”* | 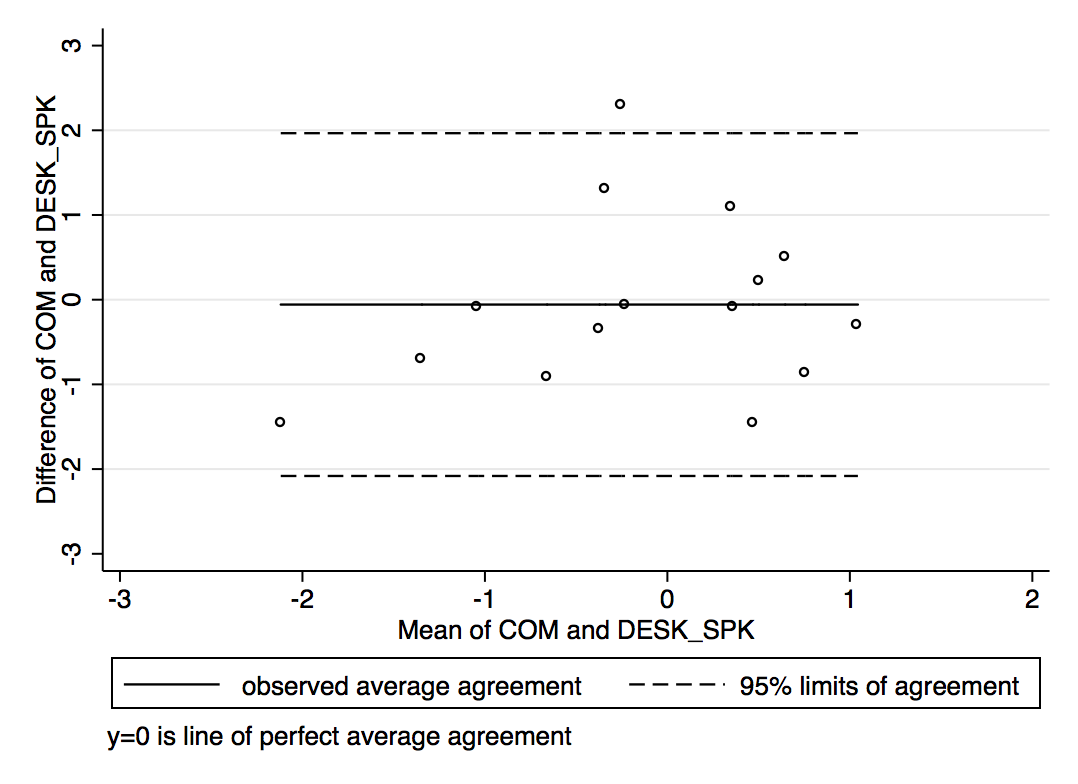  B) age 4 [95% limits of agreement: -2.08 to 1.97] |
| 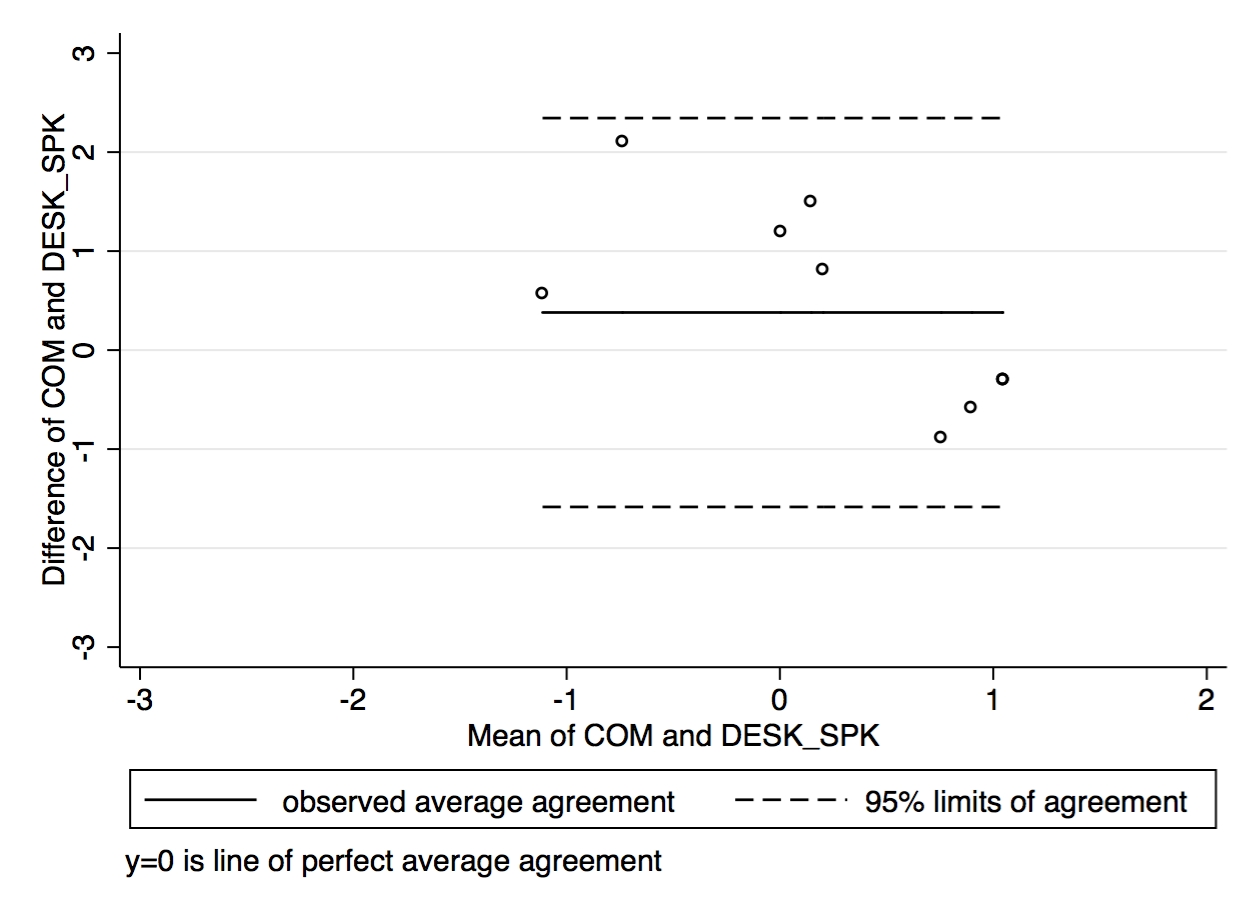  C) ages 5 & 6 [95% limits of agreement: -1.58 to 2.34] | 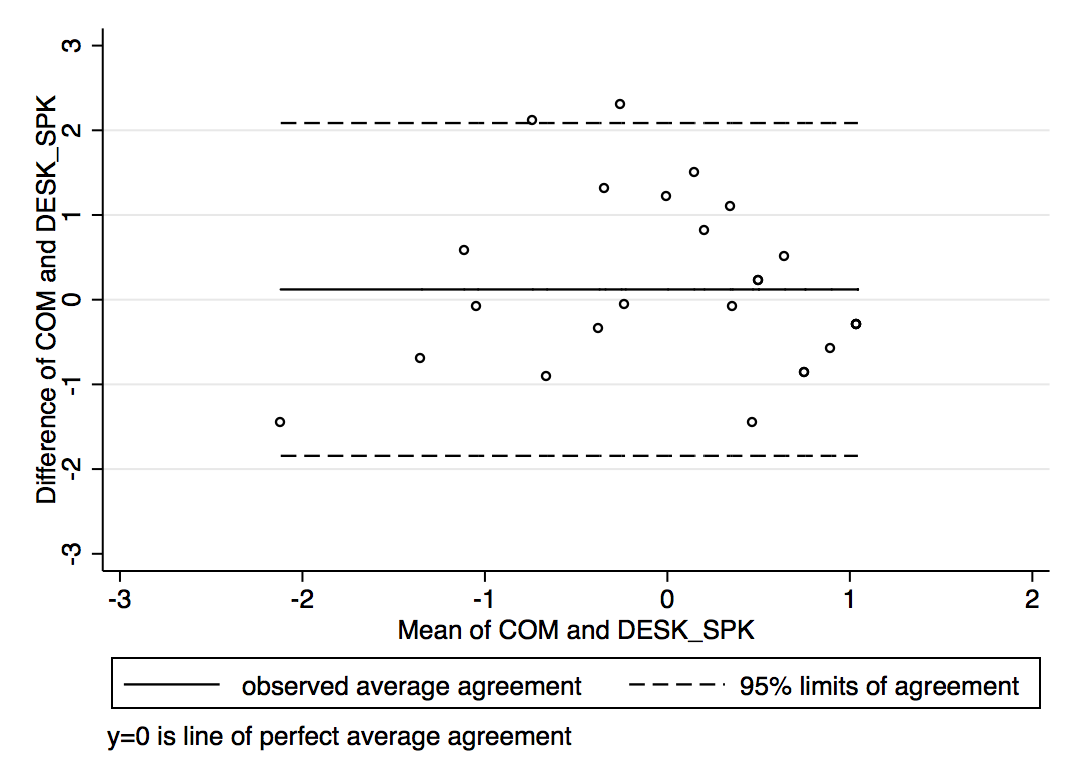  D) overall sample [95% limits of agreement -1.84 to 2.09] |
